# Engineering single-atomic ruthenium catalytic sites on defective nickel-iron layered double hydroxide for overall water splitting

**DOI:** 10.1038/s41467-021-24828-9

**Published:** 2021-07-28

**Authors:** Panlong Zhai, Mingyue Xia, Yunzhen Wu, Guanghui Zhang, Junfeng Gao, Bo Zhang, Shuyan Cao, Yanting Zhang, Zhuwei Li, Zhaozhong Fan, Chen Wang, Xiaomeng Zhang, Jeffrey T. Miller, Licheng Sun, Jungang Hou

**Affiliations:** 1 State Key Laboratory of Fine Chemicals, School of Chemical Engineering, Dalian University of Technology, Dalian, China; 2 Laboratory of Materials Modification by Laser Ion and Electron Beams (Dalian University of Technology), Ministry of Education, Dalian, China; 3 Davidson School of Chemical Engineering, Purdue University, West Lafayette, IN USA; 4 Center of Artificial Photosynthesis for Solar Fuels, School of Science, Westlake University, Hangzhou, China; 5 Department of Chemistry, School of Engineering Sciences in Chemistry, Biotechnology and Health, KTH Royal Institute of Technology, Stockholm, Sweden

**Keywords:** Electrocatalysis, Electrocatalysis, Nanoscale materials

## Abstract

Rational design of single atom catalyst is critical for efficient sustainable energy conversion. However, the atomic-level control of active sites is essential for electrocatalytic materials in alkaline electrolyte. Moreover, well-defined surface structures lead to in-depth understanding of catalytic mechanisms. Herein, we report a single-atomic-site ruthenium stabilized on defective nickel-iron layered double hydroxide nanosheets (Ru_1_/D-NiFe LDH). Under precise regulation of local coordination environments of catalytically active sites and the existence of the defects, Ru_1_/D-NiFe LDH delivers an ultralow overpotential of 18 mV at 10 mA cm^−2^ for hydrogen evolution reaction, surpassing the commercial Pt/C catalyst. Density functional theory calculations reveal that Ru_1_/D-NiFe LDH optimizes the adsorption energies of intermediates for hydrogen evolution reaction and promotes the O–O coupling at a Ru–O active site for oxygen evolution reaction. The Ru_1_/D-NiFe LDH as an ideal model reveals superior water splitting performance with potential for the development of promising water-alkali electrocatalysts.

## Abstract (web summary)

Rational design of single atom catalyst is critical for efficient sustainable energy conversion. Single-atomic-site ruthenium stabilized on defective nickel-iron layered double hydroxide nanosheets achieve superior HER and OER performance in alkaline media.

## Introduction

Hydrogen as a sustainable energy is an alternative to traditional fossil fuels to mitigate environmental problems from greenhouse gases^1,2^. Electrochemical water-splitting has been developed as an effective way to generate hydrogen fuel by use of electrocatalysts. At present, Pt- and Ir/Ru-oxide-based catalysts are the benchmark materials for hydrogen evolution reaction (HER) and oxygen evolution reaction (OER)^3–6^. However, their high cost and poor stability limit large-scale utilization. Thus, it is crucial to fabricate highly efficient catalysts for electrocatalytic applications.

Among various materials, 3*d* transition-metal-based layered double hydroxides (LDHs) containing different metals (e.g., Co, Ni, Fe, etc.) are promising electrocatalysts due to the unique lamellar structure and abundant active sites^7–9^. For example, LDHs based on Fe, Co, Ni, Zn, and Mn have been widely investigated for OER^10–16^. NiFe, NiV, and CoFe LDHs give superior OER activities in our group^17–19^. To optimize the catalytic activity of LDHs, different strategies have been developed through the regulation of morphology, defect formation, charge transfer, etc.,^20–23^. With regard to defect engineering, it is an effective approach to modulate catalytic performance. For instance, oxygen vacancies in Co_3_O_4_, sulfur vacancies in MoS_2_ and Fe vacancies in δ-FeOOH have been produced^24–28^, optimizing the electrocatalytic activity. However, it is still a challenge to control the structure of active sites in LDHs by defect engineering and develop a correlation between the defects and electrocatalytic performance.

Single atom catalysts (SACs) have emerged as a promising frontier to optimize the catalytic performance, sparking widespread interest by virtue of the appropriate coordination environment, intimate interactions of single atoms with proper supports, and quantum size effects^5,6,29–32^. Often, it is difficult to produce SACs due to facile aggregation of individual metal atoms. Interestingly, two-dimensional (2D) LDHs provide a favorable platform for the stabilization of SACs due to 2D flat facet, ultrathin thickness and high surface area^29,30^. In this regard, single atoms anchored 2D LDHs is an ideal model to maximize the OER activity, while simultaneously decreasing the content of single atoms on the support. These sites also offer a useful platform for in-depth understanding of the catalytic mechanism at an atomic level^29–32^. However, HER performance of various LDHs catalysts is rather poor owing to the large energy barrier and sluggish water dissociation kinetics in alkaline media^30–32^. Specifically, it is a challenge to stabilize single atoms on LDHs for water oxidation and reduction in the same alkaline electrolyte. Although various strategies, such as pyrolysis, wet chemistry, atomic layer deposition, etc., have been extensively explored to produce SACs with tailored requirements^33–35^, there is still no simple large-scale synthesis protocol to produce single atoms stabilized on the supports. There is a promising approach to boost the catalytic activity by the introduction of defects into the support, stabilizing single atoms due to the intimate interaction of the resulting structure^36–38^. Combining defect engineering and single atoms supported 2D LDHs, it is possible to rationally design the atomically dispersed, active single atoms stabilized on defective LDH supports for extraordinary overall water splitting performance in alkaline electrolyte.

In this work, a single-atomic-site ruthenium catalyst stabilized on defective NiFe-LDH is synthesized by a simple electrodeposition and subsequent etching procedure as the straightforward and practical approach. The combined analysis of spherical aberration-corrected transmission electron microscope and X-ray absorption fine structure (XAFS) spectroscopy reveals the existence of Ru single atoms and in-depth local atomic structures of Ni, Fe, and Ru sites. Although Ru and NiFe-LDH have been regarded to be active OER catalysts^29,30^, as-synthesized Ru_1_/D-NiFe LDH achieves a current density of 10 mA cm^−2^ at an ultralow overpotential of 18 mV and a high turnover frequency of 7.66 s^−1^ at an overpotential of 100 mV (45 times higher than that of commercial Pt/C catalyst) for HER. Inspired by the superior HER and OER performances, the assembled two-electrode cell by Ru_1_/D-NiFe LDH reaches an industrial current density of 500 mA cm^−2^ at a low cell voltage of 1.72 V for overall water splitting in alkaline media. Density functional theory (DFT) calculations suggest that Ru_1_/D-NiFe LDH optimizes the favorable regulation of H adsorption energies for HER, and promotes the O–O coupling due to the existence of Ru–O moieties. Moreover, the abundant number of active sites accelerate the water splitting kinetics, thus enhancing the intrinsic HER and OER activities. This work also establishes a promising platform for future fundamental studies into the role of isolated Ru single atoms on defective NiFe LDH nanosheets in promoting electrocatalytic performance.

## Results

### Synthesis and characterization

**Fig. 1:**
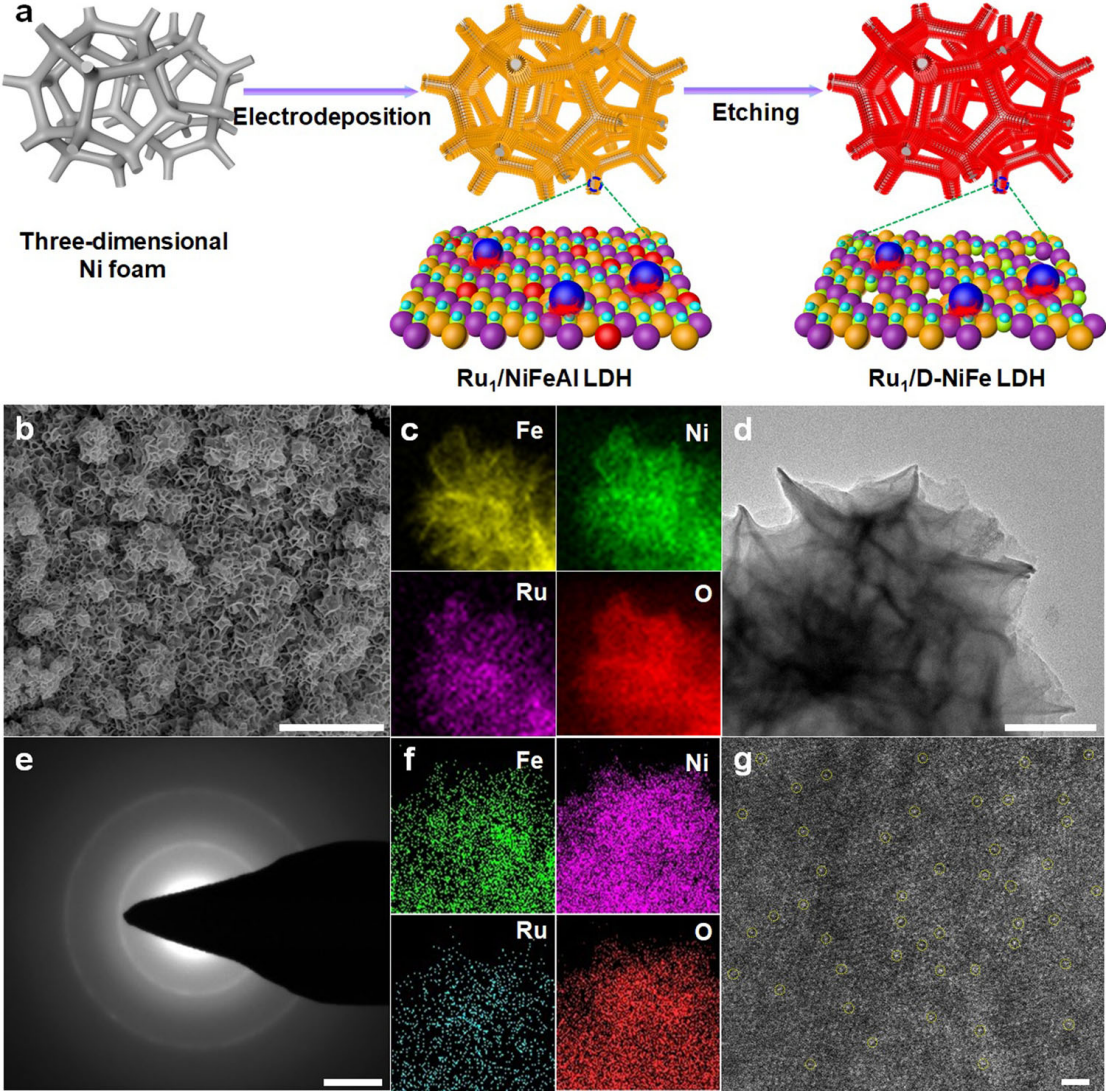
Schematic representation of synthesis and morphological characterizations. **a** Synthesis illustration and **b** SEM image, **d** TEM image, **e** SAED pattern, and **g** aberration-corrected TEM image of Ru_1_/D-NiFe LDH (isolated Ru atoms are marked with yellow circles), (cf) elemental mapping of **c** HAADF-STEM and **f** aberration-corrected TEM images of Ru_1_/D-NiFe LDH. Scale bar, **b** 1 μm, **d** 200 nm, and **g** 1 nm.

A single-atomic-site ruthenium catalyst stabilized on defective NiFe LDH (denoted as Ru_1_/D-NiFe LDH) supported on three-dimensional (3D) skeleton of nickel foam was synthesized by a facile electrodeposition and subsequent etching approach as depicted schematically in Fig. 1a. Notably, the synthetic approach was completed at room temperature and atmospheric pressure without harsh operations. Ru single atoms stabilized on NiFeAl LDH nanosheets (Ru_1_/NiFeAl LDH) were deposited onto the 3D conductive substrate by a simple electrodeposition procedure. Then, as-synthesized Ru_1_/NiFeAl LDH nanosheets were etched in alkali media, removing Al from Ru_1_/NiFeAl precursor and thus resulting into the formation of Ru single atoms integrated with defective NiFe LDH nanosheets by the precise regulation of the etching time and the content of Ru single atoms.

To shed light on the crystalline structure, X-ray diffraction (XRD) patterns verify that only a set of interplanar angles located at 44.5°, 51.8°, and 76.4° (Supplementary Fig. 1), assigned to (111), (200), and (220) planes of metallic nickel (JCPDS 04-0850), respectively, indicating the amorphous nature of NiFe LDH and Ru_1_/D-NiFe LDH nanosheets.

To identify the form of Ru single atoms dispersed on D-NiFe LDH supports, field-emission scanning electron microscopy (FE-SEM), transmission electron microscopy (TEM), high-angle annular dark-field scanning transmission electron microscopy (HAADF-STEM) and spherical aberration-corrected atomic resolution HAADF-STEM were used. As shown in Fig. 1b, SEM image of Ru_1_/D-NiFe LDH shows uniform interconnected nanosheets with a smooth surface vertically grown on 3D foam. For comparison, NiFe LDH and Ru_1_/NiFeAl LDH nanosheets were synthesized by electrodeposition, while Ru-doped NiFe LDH (denoted as NiFeRu LDH) nanosheets were prepared by hydrothermal synthesis (Supplementary Fig. 2). Based on the electrodeposition and subsequent etching treatment for various times, the typical morphological nanostructures of Ru_1_/D-NiFe LDH are 2D nanosheets after 24 h etching treatment (Supplementary Fig. 3). TEM image in Fig. 1d confirms 2D nanosheets structure of Ru_1_/D-NiFe LDH. The selected-area electron diffraction (SAED) pattern of Ru_1_/D-NiFe LDH is presented in Fig. 1e, indicating the amorphous nature with the halo-like diffraction pattern consistent with XRD. From spherical aberration corrected HAADF-STEM image, isolated Ru single atoms as bright spots are homogeneously distributed on the surface of Ru_1_/D-NiFe LDH nanosheets without any apparent nanoparticles or clusters, demonstrating the successful synthesis of single-atom electrocatalyst. The elemental mappings of Ru_1_/D-NiFe LDH are determined by HAADF-STEM and spherical aberration corrected TEM images (Fig. 1c, f), revealing the existence of Ni, Fe, Ru, and O elements and the homogeneous distribution of Ru single atoms on 2D nanosheets. SEM and TEM images, element mapping and energy-dispersive X-ray (EDX) analysis of different arrays were also conducted (Supplementary Figs. 3–10). The Ru content (1.2 wt%) of Ru_1_/D-NiFe LDH is determined by inductively coupled plasma optical emission spectrometry (ICP-OES) analysis. The atomic force microscopy (AFM) was performed to characterize the average thickness about 4.2 nm for the nanosheets (Supplementary Fig. 11). These results demonstrate that Ru single atoms stabilized on defective NiFe LDH nanosheets have been synthesized by the facile electrodeposition and subsequent etching procedure.

To unravel the chemical composition and electronic properties of the electrocatalysts, X-ray photoelectron spectroscopy (XPS) was conducted (Supplementary Fig. 12). For Ru_1_/D-NiFe LDH, the peaks located at 856.2 and 874.0 eV with two shakeup satellites are assigned to Ni 2*p*_3/2_ and Ni 2*p*_1/2_, indicating the Ni^2+^ oxidation state in Ru_1_/D-NiFe LDH^11,23^. The core-level Fe 2*p* XPS spectrum of Ru_1_/D-NiFe LDH displays the typical peaks at 712.1 and 725.7 eV, which can be indexed to Fe 2*p*_3/2_ and Fe 2*p*_1/2_ of Fe^3+^^9,21^. In comparison of NiFe LDH, there are the positive shifts of 0.5 and 0.3 eV in Ni and Fe XPS of Ru_1_/NiFe LDH and Ru_1_/D-NiFe LDH, respectively, indicating the electronic coupling of Ru single atoms and D-NiFe LDH^3,13^. In Ru 3*p* spectra, the peaks of Ru 3*p*_3/2_ and Ru 3*p*_1/2_ are located at 463.8 and 486.2 eV, intermediate between Ru (0) and Ru (IV)^39^. O 1*s* XPS core-level spectra can be divided into three spin–orbit peaks. The O 1*s* signals located at 530.3, 531.6, and 532.4 eV are assigned to metal–oxygen bond (M–O), vacancy with low oxygen coordination and adsorbed hydroxy or H_2_O, respectively^39^. Moreover, a positive shift is observed in O 1*s* XPS of Ru_1_/NiFe LDH and Ru_1_/D-NiFe LDH in comparison of NiFe LDH (Supplementary Fig. 12), indicating that Ru atom coordinates with O atoms through an intimate interaction between Ru single atoms and defective NiFe LDH support.

**Fig. 2:**
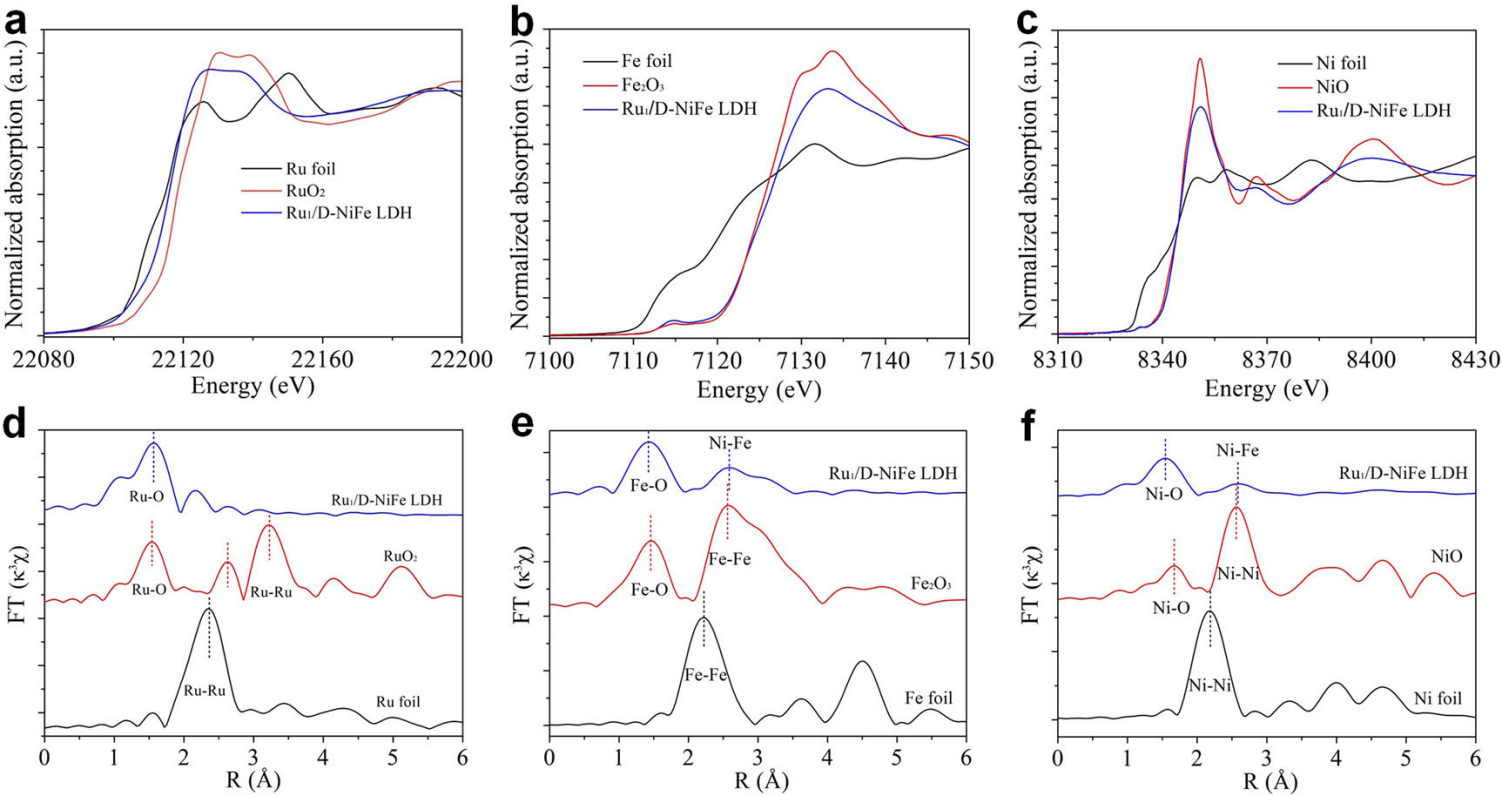
X-ray absorption spectroscopy characterizations. **a** XANES spectra at Ru K-edge of Ru_1_/D-NiFe LDH, Ru foil, and RuO_2_. **b** XANES spectra at Fe K-edge of Ru_1_/D-NiFe LDH, Fe foil, and Fe_2_O_3_. **c** XANES spectra at Ni K-edge of Ru_1_/D-NiFe LDH, Ni foil, and NiO. **d**–**f** Fourier-transform EXAFS spectra from **a**–**c**.

To determine the valance states and local coordination structures of Ru_1_/D-NiFe LDH at the atomic level, X-ray absorption near-edge structure (XANES) spectroscopy and extended X-ray absorption fine structure (EXAFS) spectroscopy were performed. The Ru K-edge XANES spectra of Ru_1_/D-NiFe LDH in Fig. 2a shows that the edge energy is between Ru foil and RuO_2_, demonstrating a cationic environment. In the Fourier-transform EXAFS (FT-EXAFS) spectra in Fig. 2d, Ru_1_/D-NiFe LDH has a first shell Ru–O peak at 1.56 Å (phase uncorrected distance) and a weak Ru–O–M (M = Ni or Fe) in the higher shells. Compared to Ru foil and RuO_2_, there is no characteristic peak for Ru–Ru scattering in Ru_1_/D-NiFe LDH, which is consistent with the analysis of aberration-corrected TEM image of atomically dispersed Ru atoms on 2D LDH nanosheets^29,40^. The analysis of coordination configuration was conducted by model-based EXAFS fitting. The coordination number of Ru–O in first coordination sphere of Ru_1_/D-NiFe-LDH is estimated to be 3.7, implying the existence of coordinatively unsaturated RuO_4_ sites (Supplementary Table 1 and Supplementary Figs. 13 and 14). XANES simulation on Ru_1_/D-NiFe-LDH presents that the white line and post-edge features are well reproduced in experimental and simulated spectra (Supplementary Fig. 15). All above results indicate that atomically dispersed Ru is successfully immobilized on D-NiFe LDH nanosheets by coordianting with O atoms. As shown in the Fe K-edge XANES spectra in Fig. 2b, a pre-edge energy of 7114.8 eV for Ru_1_/D-NiFe LDH, identical to that of Fe_2_O_3_, suggests the presence of Fe^3+^, which is in agreement with the XPS analysis. A slightly higher pre-edge intensity than that of Fe_2_O_3_ likely indicates a distorted octahedral coordination geometry of the Fe^3+^ sites in Ru_1_/D-NiFe LDH^41^. The R-space shows two prominent coordination peaks at 1.44 and 2.58 Å (phase uncorrected distance), which can be assigned to the Fe–O peak and Fe–Ni/Fe peak. Moreover, the average Fe–Ni/Fe distance in Ru_1_/D-NiFe LDH is slightly shorter than that of NiFe LDH, indicating the presence of coordinatively unsaturated sites and structure distortion around Fe center^40,41^. As for Ni K-edge, the pre-edge peak of Ru_1_/D-NiFe LDH (8333.4 eV) is similar to that of the NiO reference (Fig. 2), indicating the presense of Ni^2+^, which is in accordance with the analysis of XPS spectra. The similar change of Ni or Fe EXAFS was obtained with the precense of abundant metal defect sites in Ru_1_/D-NiFe LDH nanosheets^26,42,43^. Based on the EXAFS analysis, the abundant number of defects in the LDH nanosheets could play an important role in stabilizing Ru atoms on LDH support.

### Electrocatalytic performance for HER

**Fig. 3:**
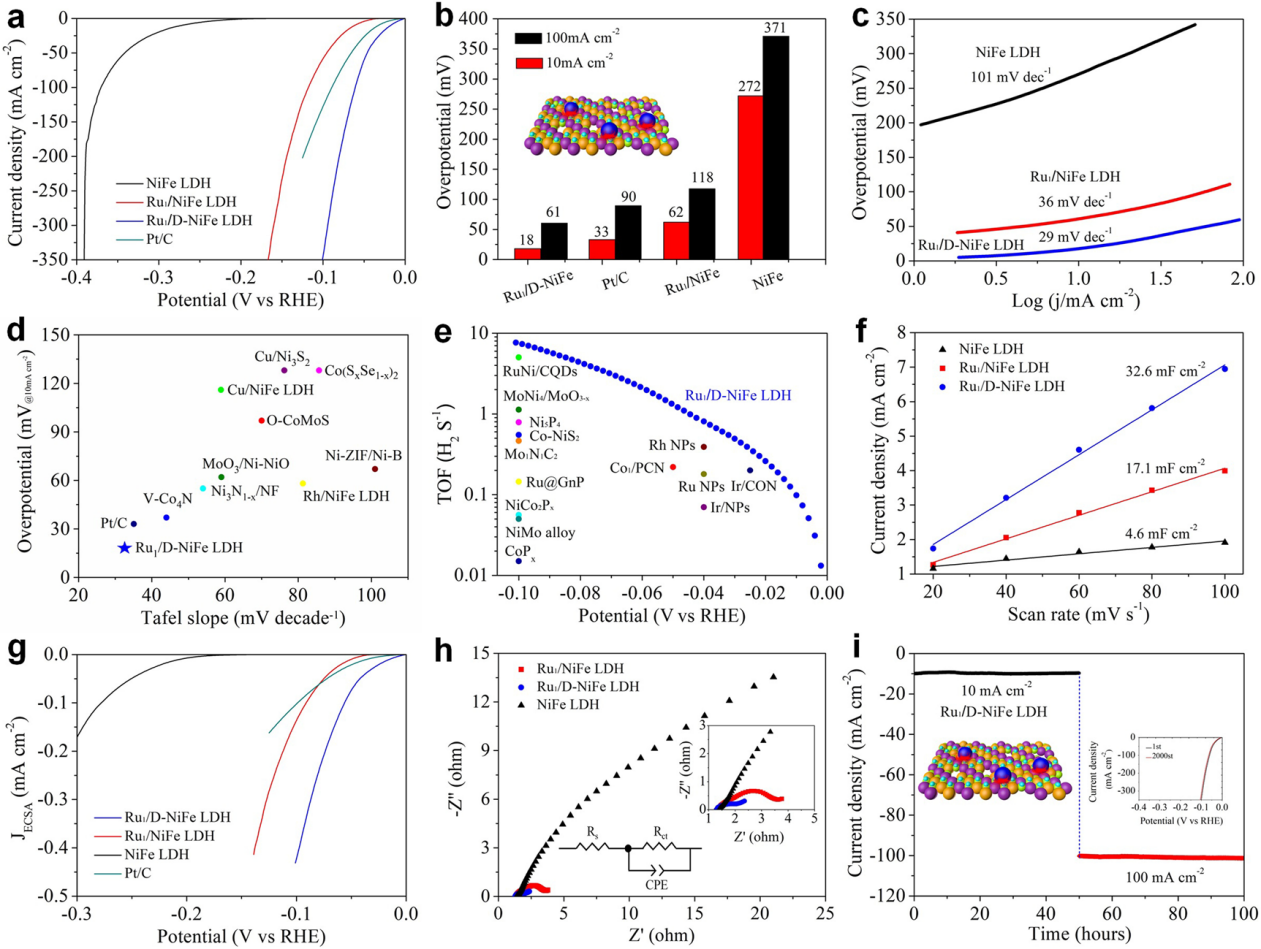
HER catalytic performance. **a** HER polarization curves, **b** overpotentials at typical current densities, **c** Tafel slopes of various LDHs, **d** comparison of merit with respect to both kinetics (Tafel slope) and activity (the overpotential at 10 mA cm^−2^), **e** TOF values of Ru_1_/D-NiFe LDH (blue dots), together with reported HER electrocatalysts at typical overpotentials, **f** double-layer capacitances (*C*_dl_), **g** polarization curves with the current normalized to ECSA of NiFe LDH, Ru_1_/NiFe LDH, and Ru_1_/D-NiFe LDH, **h** electrochemical impedance spectroscopy, **i** time-dependent current density curves of Ru_1_/D-NiFe LDH at −0.018 and −0.061 V vs. RHE. Inset is LSV curves before and after 2000 cycles.

Owing to the kinetically sluggish electrocatalytic HER by NiFe LDH, it is important to optimize the HER activity. The electrocatalytic HER performance of all electrocatalysts by linear sweep voltammetry (LSV) was evaluated using a typical three-electrode system in nitrogen-saturated 1 M KOH electrolyte. As shown in Fig. 3a, b, Ru_1_/D-NiFe LDH with only 1.2 wt% of Ru single atoms has the catalytically most active polarization curve with a near zero onset potential. Ru_1_/D-NiFe LDH delivers the overpotentials of 18 and 61 mV to reach the current densities of 10 and 100 mA cm^−2^, which are lower than the benchmark catalysts, 33 and 90 mV for Pt/C catalyst, and 272 and 371 mV for NiFe LDH, indicating that Ru single atoms stabilized on D-NiFe LDH nanosheets can significantly improve the electrocatalytic performance. In comparison to Ru_1_/D-NiFe LDH, the HER electrocatalytic activities of Ru single atoms supported on NiFe LDH (Ru_1_/NiFe LDH), NiFeAl LDH (Ru_1_/NiFeAl LDH) and defective NiFe LDH (D-NiFe LDH) are low (Supplementary Fig. 16), highlighting the important role of the defects and Ru single atoms on NiFe LDH surface. The difference between Ru_1_/D-NiFe LDH and Ru_1_/NiFe LDH suggests that defect-rich NiFe LDH has a stronger interaction with Ru single atoms than NiFe LDH, leading to a significant enhancement in the HER performance. By tuning the etching time and the amount of Ru single atoms in Ru_1_/D-NiFe LDH, the catalytic performance can be optimized (Supplementary Fig. 17). Tafel plots were derived from the polarization curves to capture deeper insight of the electrochemical reaction kinetics (Fig. 3c). The Tafel slope of Ru_1_/D-NiFe LDH is as low as 29 mV dec^−1^, which is lower than those of Ru_1_/NiFe LDH (36 mV dec^−1^) and NiFe LDH (101 mV dec^−1^), indicating that the reaction pathways follow the Volmer–Tafel mechanism and the chemical recombination of adsorbed H is the rate-determining step^35^. Moreover, the exchange current density (*j*_0_) determined by extrapolating the Tafel plot of Ru_1_/D NiFe LDH is estimated at 2.6 mA cm^−2^ (Supplementary Fig. 18), which is better than those of other catalysts, revealing the excellent inherent electrocatalytic activity. With regard to low overpotential and Tafel slope, the apparent merits of Ru_1_/D-NiFe LDH are significantly better than those reported for commercial Pt/C and most HER catalysts (Fig. 3d and Supplementary Table 2). Interestingly, an impressive mass activity, 14,650 A g_metal_^−1^ of Ru_1_/D-NiFe LDH was achieved at the overpotential of 100 mV, which is ~7 times higher than that of Ru_1_/NiFe LDH (2420 A g_metal_^−1^), and ~45 times higher than that of the Pt/C catalyst (320 A g_metal_^−1^). The turnover frequency (TOF) was calculated (Fig. 3e), assuming all Ru are presented as active sites to quantify the catalytic efficiency. The TOF of Ru_1_/D-NiFe LDH is 7.66 s^−1^ at the overpotential of 100 mV, which is ~6 times higher than that of Ru_1_/NiFe LDH (1.27 s^−1^), and ~24 times higher than that of Pt/C catalyst (0.32 s^−1^) as well as higher than those of most reported catalysts (Supplementary Table 3), implying the highly efficient utilization of Ru single atoms on defective LDH support. To evaluate the active surface area of the electrocatalysts, the electrochemical surfaces areas (ECSA) were obtained by double-layer capacitance (*C*_dl_) in non-Faradaic potential region^22–35^. Ru_1_/D-NiFe LDH has the highest *C*_dl_ value of 32.6 mF cm^−2^, which is 1.9- and 7.1-fold times higher than those of Ru/NiFe LDH and NiFe LDH, respectively (Fig. 3f and Supplementary Fig. 19). Especially, the current densities of NiFe LDH, Ru_1_/NiFe LDH, Ru_1_/D-NiFe LDH, and commercial Pt/C were normalized to ECSA (Fig. 3g and Supplementary Figs. 20, 21), demonstrating the highest instrinsic activity of Ru_1_/D-NiFe LDH. Electrochemical impedance spectroscopy (EIS) measurement was performed to get insight into the kinetics of charge transfer. Based on Nyquist plots, the charge transfer resistance (*R*_ct_) of Ru_1_/D-NiFe LDH is smaller than those of other electrocatalysts, demonstrating a facilitated HER charge transfer kinetics and a fast Faradaic reaction process at the interface between the catalyst and the electrolyte (Fig. 3h and Supplementary Fig. 22). Thus, the synergistic effect of the defects and Ru single atoms faciliates the charge transport and increases the number of active sites. To identify the durability of the catalyst, LSV curves before and after 2000 cycles tests show little change (Fig. 3i). The time-dependent current density curves of Ru_1_/D-NiFe LDH were recorded, delivering 10 and 100 mA cm^−2^ at −0.018 and −0.061 V vs. RHE for 100 h. Negligible change of current density is also observed in Fig. 3i, indicating the excellent cycling and long-term stability. The extraordinary stability is ascribed to the unique structures of Ru single atoms integrated NiFe LDH nanosheets with abundant defect sites, stabilizing Ru single atoms at atomic level and facilitating the charge transfer in Ru_1_/D-NiFe LDH. Afterwards, hydrogen generation was analyzed (Supplementary Fig. 23), indicating that Faradaic efficiency (FE) of Ru_1_/D-NiFe LDH is close to 100% for real water splitting.

### Electrocatalytic performance for OER

**Fig. 4:**
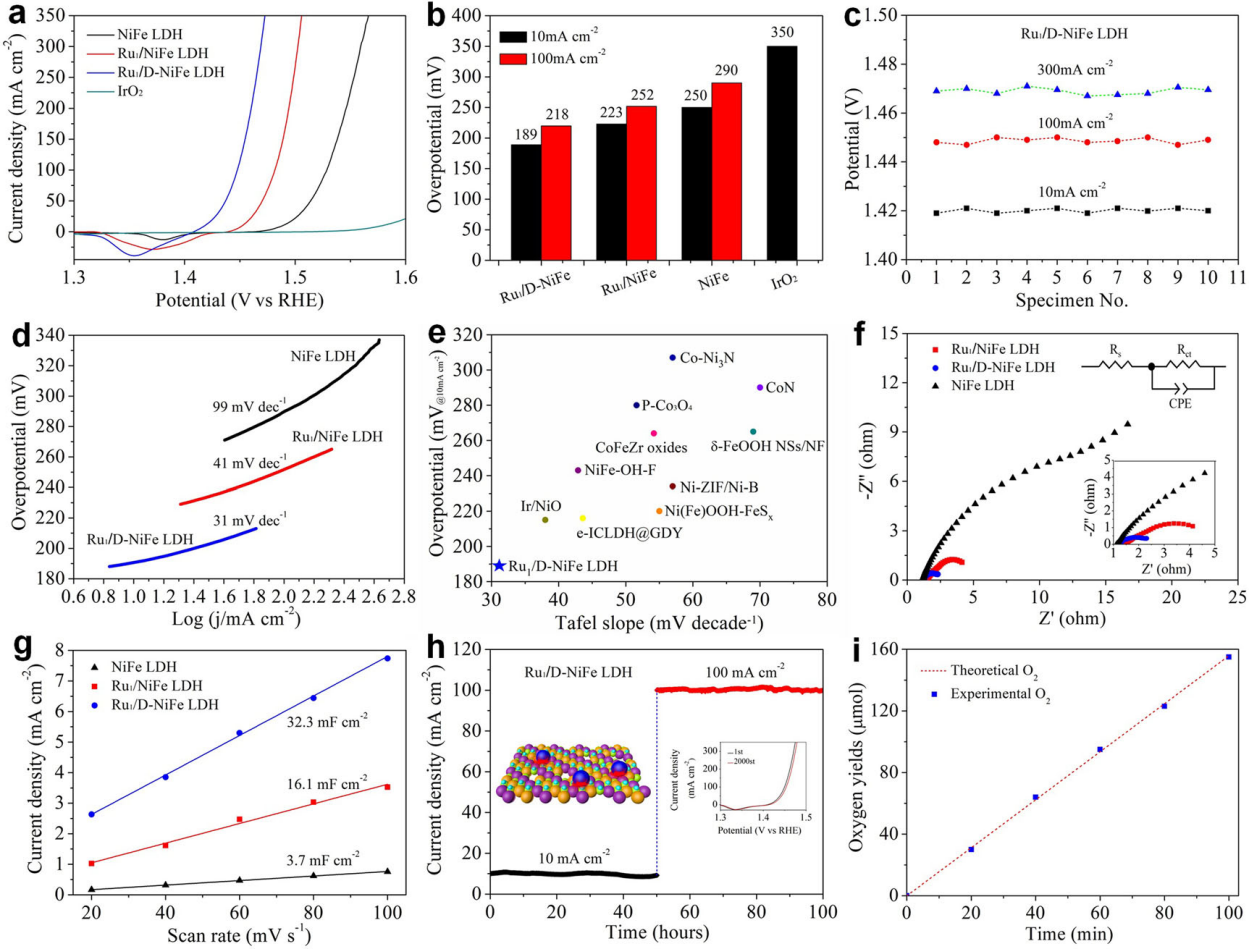
OER catalytic performance. **a** OER polarization curves, **b** overpotentials at typical current densities, **c** potentials at current densities of 10, 100, and 300 mA cm^−2^ for ten Ru_1_/D-NiFe LDH electrodes, **d** Tafel slopes of various LDHs, **e** comparison of merit with respect to both kinetics (Tafel slope) and activity (the overpotential at 10 mA cm^−2^), **f** electrochemical impedance spectroscopy, **g** double-layer capacitances (*C*_dl_) of of NiFe LDH, Ru_1_/NiFe LDH, and Ru_1_/D-NiFe LDH, **h** time-dependent current density curves of Ru_1_/D-NiFe LDH at 1.419 and 1.448 V vs. RHE. Inset is LSV curves before and after 2000 cycles. **i** The amount of gas theoretically calculated and experimentally measured vs. time by use of Ru_1_/D-NiFe LDH.

The OER performace of the electrocatalysts was also evaluated in oxygen-saturated 1 M KOH solution. For Ru_1_/D-NiFe LDH, a sharp increase in the anodic current response was observed at an onset potential of 1.41 V vs. RHE. Strikingly, Ru_1_/D-NiFe LDH increases dramatically after the onset potential with small overpotentials of 189 and 220 mV at 10 and 100 mA cm^−2^, which is lower than those of NiFe LDH (250 and 290 mV) and commericial IrO_2_ (350 mV) in Fig. 4a, b, demonstrating remarkable OER activity. What is the most significant is that a high current density up to 300 mA cm^−2^ can be achieved at 1.47 V vs. RHE for Ru_1_/D-NiFe LDH owing to efficient charge transfer, large electrochemical surface area and unique architecture as well as the synergistic effect of Ru single atoms and the defective support. In comparison of Ru_1_/D-NiFe LDH, Ru_1_/NiFe LDH, Ru_1_/NiFeAl LDH and D-NiFe LDH present the inferior OER electrocatalytic activities (Supplementary Fig. 24). Compared to Ru-doped NiFe (NiFeRu) LDH by hydrothermal process^44,45^, Ru_1_/NiFe LDH shows high OER activity (Supplementary Fig. 25), demonstrating this electrodeposition approach is a promising strategy to fabricate the integtration of single atoms and 2D nanomaterials. The superior Ru_1_/D-NiFe LDH is achieved by regluating the etching time and the amount of Ru single atoms in Ru_1_/D-NiFe LDH (Supplementary Fig. 25). With regard to the reproducibility, ten Ru_1_/D-NiFe LDH electrodes were synthesized by the same approach. There is no big change upon the potentials of 1.419, 1.448 and 1.469 V vs. RHE to deliver the current densities of 10, 100, and 300 mA cm^−2^, as shown in Fig. 4c, demonstrating the excellent reproducibility. In addition to the low overpotential and high current density, the Tafel plot in Fig. 4d shows that Ru_1_/D-NiFe LDH has a small slope of 31 mV dec^−1^, which is lower than those of Ru_1_/NiFe LDH (41 mV dec^−1^) and NiFe LDH (99 mV dec^−1^), respectively, suggesting favorable OER kinetics in alkaline electrolyte. The combined merits of Ru_1_/D-NiFe LDH including low overpotentials and Tafel slopes (Fig. 4e), are superior to commercial IrO_2_ and most reported OER catalysts (Supplementary Table 4)^15,46–54^. Particularly, the mass activity, 11,980 A g_metal_^−1^ of Ru_1_/D-NiFe LDH was obtained at the overpotential of 240 mV, suggesting that Ru single atoms supported on defective NiFe LDH nanosheets can dramatically maximize the OER activity.

From Nyquist plots, Ru_1_/D-NiFe LDH exhibits a smaller semicircle diameter than others, implying the faster charge transfer between the electrodes and the electrolyte (Fig. 4f). To investigate the origin of the enhancement of OER performance, a large *C*_dl_ value, 32.3 mF cm^−2^ was obtained for Ru_1_/D-NiFe LDH, which is higher than those of Ru_1_/NiFe LDH and NiFe LDH (Fig. 4g and Supplementary Fig. 26), indicating an abundant number of active sites. Ru_1_/D-NiFe LDH also has impressive durability in alkaline media (Fig. 4h). The amount of oxygen was measured in comparison of actual quantity against theoretical quantity at differernt reaction time (Fig. 4i), revealing that the FE of Ru_1_/D-NiFe LDH is 99.6% during OER process and the observed catalytic current originates exclusively from water oxidation. Accordingly, the intimate interaction between Ru single atoms and defective NiFe LDH nanosheets is beneficial to the OER enhancement of Ru_1_/D-NiFe LDH.

### Electrocatalytic performance for overall water splitting

**Fig. 5:**
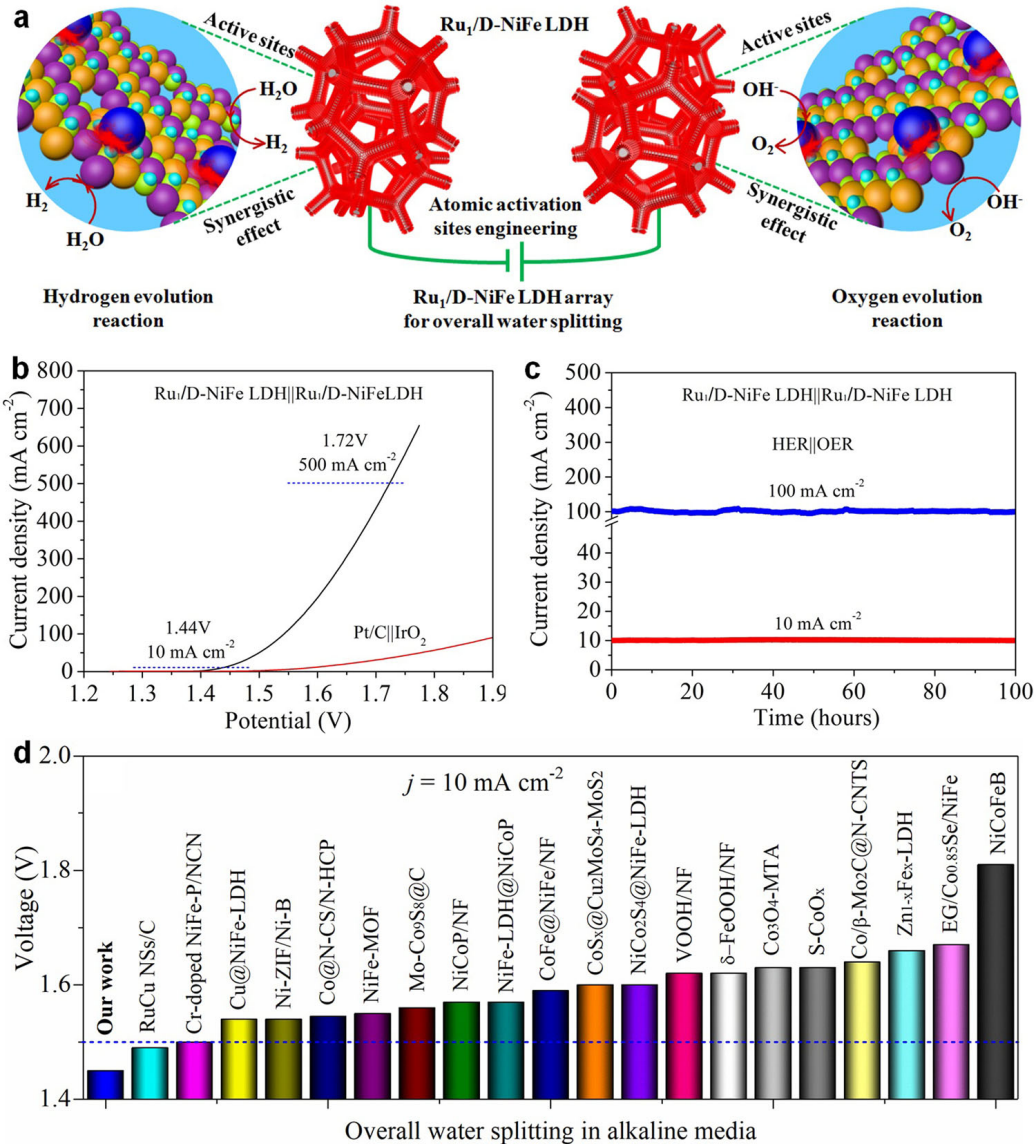
Electrocatalytic performance for overall water splitting. **a** Schematic diagram of water splitting in a two-electrode configuration, (**b**) polarization curves by two-electrode system, **c** chronoamperometric test of Ru_1_/D-NiFe LDH with current densities of 10 and 100 mA cm^−2^ at 1.44 and 1.54 V, and **d** comparison of the cell voltages at 10 mA cm^−2^ for Ru_1_/D-NiFe LDH with reported bifunctional electrocatalysts.

Inspired by the superior hydrogen and oxygen evolution performance of Ru_1_/D-NiFe LDH, a two-electrode configuration electrolyzer (Ru_1_/D-NiFe LDH‖Ru_1_/D-NiFe LDH) for overall water splitting (Fig. 5a) was assembled by Ru_1_/D-NiFe LDH as both the anode and cathode. The polarization curves of as-prepared Ru_1_/D-NiFe LDH‖Ru_1_/D-NiFe LDH and Pt/C‖IrO_2_ as the benchmark catalysts are displayed in Fig. 5b. Ru_1_/D-NiFe LDH only requires the low cell voltages of 1.44 and 1.54 V to reach current densities of 10 and 100 mA cm^−2^_,_ respectively, which is even better than Pt/C‖IrO_2_ and most reported bifunctional electrocatalysts (Fig. 5d and Supplementary Table 5). Particularly, Ru_1_/D-NiFe LDH as a quintessence drives the typical two-electrode cell to the industrially required current density as high as 500 mA cm^−2^ at an ultralow cell voltage of 1.72 V. The overpotential at 500 mA cm^−2^ by Ru_1_/D-NiFe LDH is lower than most of reported bifunctional catalysts (175 mA cm^−2^ at 1.8 V for NiVIr-LDH||NiVRu-LDH^42^, 500 mA cm^−2^ at 1.735 V for NiMoN||NiMoN@NiFeN^55^, 190 mA cm^−2^ at 1.7 V for Rh/NiFeRh-LDH||Rh/NiFeRh-LDH^56^, 50 mA cm^−2^ at 1.76 V for P-Co_3_O_4_||P-Co_3_O_4_^24^, etc.), indicating promising potential for industrial overall water splitting application.

Significantly, as-assembled Ru_1_/D-NiFe LDH‖Ru_1_/D-NiFe LDH electrodes presented long-term stability by time-dependent current density curves. There is no obvious degradation of the current densities of 10 and 100 mA cm^−2^ at constant potentials of 1.44 and 1.54 V over 100 h, indicating the good durability. After long-term electrocatalysis, there is a positive shift of two peaks at 856.5 and 874.3 eV for the Ni 2*p* XPS (Supplementary Fig. 27), confirming the formation of high valence state of Ni^3+^ from the oxidation of Ni^2+^^23,57–61^. In addition, a new peak located at 869.1 eV occured in the XPS of Ni 2*p*, demonstrating the existence of oxyhydroxides as the active sites after long-term OER. However, there is little change in the morphologies and components of Ru_1_/D-NiFe LDH after HER and OER by use of SEM and element mappings (Supplementary Figs. 28–30). Additionally, the atomic dispersion of Ru single atoms in D-NiFe LDH after long-tern OER is maintained (Supplementary Fig. 31). The combined analysis of these results demonstrates the potential of Ru single atoms stabilized on defective NiFe-LDH as a promising candidate towards overall water splitting.

To gain in-depth insights into the structure evolution and electrocatalytic mechanism during OER process, in-situ Raman spectra was used from open circuit voltage (OCV) to 1.7 V vs. RHE in 1 M KOH. When the applied potential was higher than 1.4 V vs. RHE, a pair of characteristic Raman peaks appeared at 447 and 557 cm^−1^, which can be assigned to the Ni^3+^–O *e*_g_ bending and *A*_1g_ stretching vibrations of γ-NiOOH (Supplementary Fig. 32a), indicating the pristine structure transformed to the oxyhydroxides under oxygen evolution potential^62–64^. Interestingly, when the potential decreases from 1.7 to 1.2 V vs. RHE, the peak of γ-NiOOH disappeared, suggesting the oxyhydroxides transformed back to LDH (Supplementary Fig. 32b). In brief, these results reveal that the reversible transformation between LDH and the oxyhydroxides are the real active species.

### First-principles calculations

DFT calculations were performed to identify the active sites in Ru_1_/D-NiFe LDH. The Gibbs free energy for each elementary reaction step in HER and OER were explored. The hydrogen absorption energy (∆*G*_H*_) of adsorbed H is a key descriptor for evaluating the HER performance^3,29,65^. The adsorption structures of H at Ni and Fe sites in NiFe LDH, and Ru site on Ru_1_/D-NiFe LDHs were modeled (Supplementary Fig. 33a–c). Compared with ∆*G*_H*_ of adsorbed H at Ni site (1.53 eV) and Fe site (1.16 eV) in NiFe LDH (Supplementary Fig. 33d), H adsorption at the Ru site on Ru_1_/D-NiFe LDH has a lower ∆*G*_H*_ value of 0.25 eV, indicating that the Ru site has more favorable enthalpy of hydrogen adsorption and simultaneous decrease in the thermodynamic barriers for hydrogen production. The DFT results demonstrate the importance of the synergistic effect between the isolated Ru single atoms and defective LDH for high hydrogen evolution rates in alkaline medium.

**Fig. 6:**
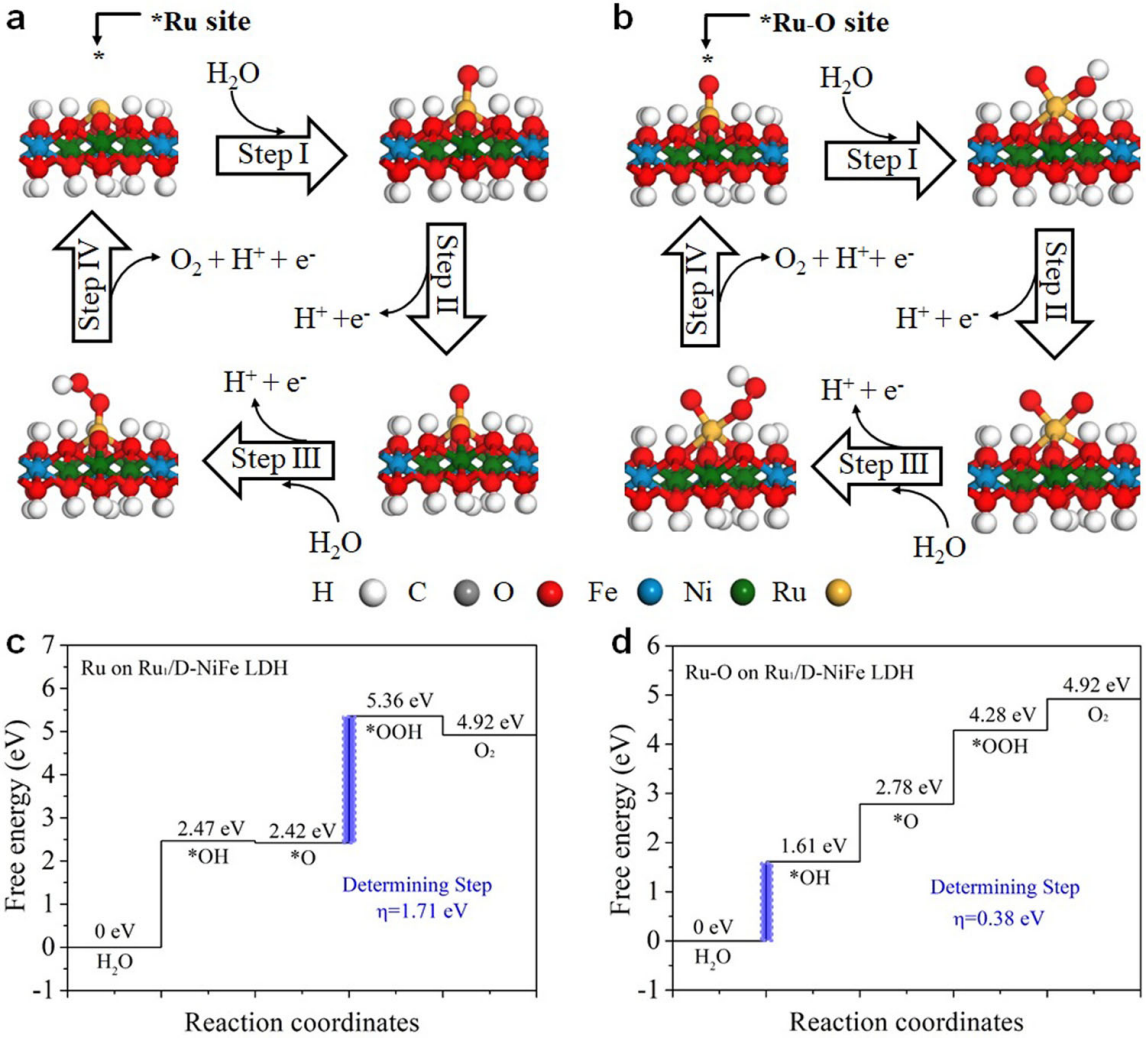
DFT calculations. **a**, **b** Schematic illustration of the proposed OER mechanism and **c**, **d** Gibbs free energy diagram for **a**, **c** Ru and **b**, **d** Ru–O sites on Ru_1_/D-NiFe LDH. The lavender box step is the rate-determining step.

DFT modeling of the water oxidation mechanism involving four concerted proton–electron transfer steps was also analyzed in alkaline medium. As shown in Fig. 6, each elementary reaction step of Gibbs free energy was calculated. The edge sites of Ni and Fe in NiFe LDH were selected as the active site for OER. For NiFe LDH, the transition from O* to OOH* and the formation O* from OH* are the rate-determining steps for Fe sites and Ni sites, respectively (Supplementary Figs. 34, 35)^5^. The large Gibbs free energies of the rate-determining step for Fe and Ni sites lead to low O_2_ rates, presenting the sluggish OER kinetics. The DFT results suggest that the rate-determining step for Ru site on Ru_1_/D-NiFe LDH is the transition O* to OOH*, with the overpotential of 1.71 eV (Fig. 6a). Surprisingly, the Gibbs free energy of Ru sites is higher than those of Fe and Ni sites in NiFe LDH, which is not in agreement with the electrocatalytic performance of Ru_1_/D-NiFe LDH. From previous reports, M–O (M = Ir, Ru) was shown as the likely reaction site during OER^5,30^. Herein, Ru–O moiety on Ru_1_/D-NiFe LDH (Fig. 6b) was proposed as the active site and modeled for the four elementary OER reaction steps. The rate-determining step for Ru–O site on Ru_1_/D-NiFe LDH is the formation of OH*. With this active site structure, the overpotential of the rate-determining step for Ru–O site decreased to 0.38 eV, which is even lower that those of Fe–O and Ni–O sites, expediting the OER kinetics. Thus, both experiment and theoretical simulation confirm that the synergetic effect between Ru single atoms and defective NiFe LDH is beneficial to accelerate the reaction kinetics and thus promote the enhancement of electrocatalytic performance of Ru_1_/D-NiFe LDH.

## Discussion

In summary, a simple and scalable electrocatalytic synthesis strategy is described for stabilization of large number of single atom ruthenium sites on defective NiFe LDH. Based on the precise regulation of local coordination environments of the active sites and the existence of the defects, Ru_1_/D-NiFe LDH achieves superior HER and OER performance in alkaline media with low overpotential, high current density and long-term durability. The well-defined structures of the catalysts also allow for fundamental investigation into the reaction steps and kinetics of the HER and OER reactions. For example, DFT calculations reveal that Ru_1_/D-NiFe LDH promotes the favorable regulation of H adsorption energies for HER, and facilitates the O–O coupling for OER. For the OER, the Ru–O moiety is proposed as active site for high rates of O_2_ formation. This work not only develops a simple and practical strategy for the synthesis of Ru_1_/D-NiFe LDH, but also theoretically/experimentally confirms the pivotal roles of single atoms in unexpectedly optimizing electrocatalytic activity, opening up new opportunities for efficient and stable electrocatalysts with potential for the development of an improved commercial water splitting process.

## Methods

### Synthesis of Ru_1_/D-NiFe LDHs

The electrolyte was containing 0.12 M Ni(NO_3_)_2_·6H_2_O, 0.12 M Fe(NO_3_)_3_·9H_2_O, 0.001 M Al(NO_3_)_3_·9H_2_O and 0.01–0.06 M RuCl_3_·*x*H_2_O. A constant potential electrodeposition was conducted at −1.0 V vs. Ag/AgCl for a certain time. Ru single atoms stabilized NiFeAl LDH (denoted as Ru_1_/NiFeAl LDH) as the precursor was fabricated by electrodeposition approach. Then, Ru_1_/NiFeAl LDH supported on nickel foam was immersed in 5 M NaOH solution under continuous stirring for various times (12, 24, and 36 h). After alkaline etching treatment, Ru single atoms integrated defective NiFe LDH (denoted as Ru_1_/D-NiFe LDH) nanosheets were obtained. In comparison, Ru_1_/NiFe LDH was synthesized by the same procedure of Ru_1_/D-NiFe LDH without alkaline etching, by use of 0.12 M Ni(NO_3_)_2_·6H_2_O, 0.12 M Fe(NO_3_)_3_·9H_2_O, and 0.01–0.06 M RuCl_3_·*x*H_2_O. NiFe LDH was fabricated by the same procedure of Ru_1_/D-NiFe LDH, without use of RuCl_3_·*x*H_2_O and alkaline etching.

### Structural characterization

Powder XRD patterns were characterized using X-ray diffractometer (Japan Rigaku Rotaflex) by Cu K_α_ radiation (*λ* = 1.5418 Å). SEM tests were recorded on Nova NanoSEM 450. TEM and HAADF-STEM images were performed on FEI TF30. Spherical aberration-corrected TEM images were characterized on a JEM ARM200F thermal-field emission microscope with a probe spherical aberration corrector. XPS data was tested by a model of ESCALAB250. Inductively coupled plasma-optical emission spectrometer (ICP-OES) was characterized on PerkinElmer AVIO 500. In-situ Raman experiments were conducted with a Raman spectrometer (Thermo Fisher, DXR Microscope) with a ×50 visible objective. The wavenumber of the excitation light source was 532 nm. Atomic force microscope (AFM, Bruck Dimension Icon) was utilized to analyze the thickness of the product. X-ray absorption fine structure spectra (XAFS) of Ni, Fe, and Ru K-edge were collected at BL07A1 beamline of National Synchrotron Radiation Research Center (NSRRC). The data were collected in fluorescence mode using a Lytle detector.

### Electrochemical measurements

The electrochemical measurements were conducted by a standard three-electrode cell with the connection of an electrochemical workstation. As-synthesized LDH-based electrode was employed as the working electrode. A graphite rod was applied as the counter electrode. Hg/HgO electrode was utilized as the reference electrode. The potentials were converted to RHE by the equation, *E*_RHE_ = *E*_Hg/HgO_ + 0.059 pH + 0.098 V. The geometric surface area of the catalysts supported Ni foam is 1 cm^2^, corresponding the mass loading of Ru_1_/D-NiFe LDHs (2 mg cm^−2^). In comparison, 20 wt% Pt/C and IrO_2_ inks were drop-cast onto Ni foam, producing Pt/C and IrO_2_ electrodes for electrochemical tests. The HER and OER polarization curves were measured by a LSV approach with a sweeping rate of 1 mV s^−1^ in nitrogen- and oxygen-saturated 1 M KOH media at 25 ^o^C. EIS was performed within the frequency range from 100 kHz to 0.1 Hz. With regard to the measurement of FE, the gaseous products were conducted by gas chromatography (Shimadzu, GC-2014). For determination of FE, the efficiency of HER or OER catalysts is defined as the ratio of the amount of experimentally determined hydrogen or oxygen to that of the theoretically expected hydrogen or oxygen from the HER or OER reaction in 1 M KOH solution. The calculation of mass activity, TOF measurement and XANES simulation were presented (Supplementary Notes 1–3).

### First-principle calculations

All DFT calculations were performed by the Vienna ab initio simulation package (VASP)^66^. The projector augmented wave pseudopotentials and the generalized gradient approximation parameterized by Perdew–Burke–Ernzerhof (GGA-PBE) for exchange-correlation functional^67^. The core electrons were descripted by the Projector-augmented wave (PAW) technology. The Brillouin zones of the supercells were sampled by 1 × 2 × 3 uniform *k* point mesh^66^. With fixed cell parameters, the model structures were fully optimized using the convergence criteria of 10^−5^ eV for the electronic energy and 10^−2^ eV/Å for the forces on each atom and the plane wave cutoff was set to 400 eV. The supercells dimension in *y* and *z* was 10.14 and 6.44 Å, respectively. The vacuum region in the *z* direction was adopted large than 20 Å. Both spin-polarized and spin-unpolarized computations were performed. Considering the strong *d*-electron correlation effects for Fe and Ni, DFT + *U* method was used in this work with *U* = 3.9 eV and *J* = 0 eV for Fe and *U* = 2.9 eV and *J* = 0 eV for Ni. and *U* = 3.4 eV and *J* = 0 eV for Ru.

The Gibbs free energy of the intermediates for HER and OER process, that is, *H, *OH, *O, and *OOH, can be calculated as

$$\Delta G={E}_{{\mathrm {ads}}}+\Delta {E}_{{\mathrm {ZPE}}}-T\Delta S-\Delta G(pH)+eU$$

Here *E*_ads_ is the adsorption energy of intermediate, ∆*E*_ZPE_ is the zero point energy difference between the adsorption state and gas state, *T* is the temperature (300 K), ∆*S* is the entropy various between the adsorption and gas phase.

The intermediates adsorption energy *E*_ads_ for *H, *OH, *O and *OOH can be used DFT ground state energy calculated as^68^

1 $$\Delta {E}_{\ast {{{MH}}}}=E(\ast {{MH}})-E(\ast )-1/2E({H}_{2})$$

2 $$\Delta {E}_{\ast {{OOH}}}=E(\ast {{OOH}})-E(\ast )-(2{E}_{{{{H}}}_{2}{{O}}}-3/2{E}_{{{{H}}}_{2}})$$

3 $$\Delta {E}_{\ast {{O}}}=E(\ast {{O}})-E(\ast )-({E}_{{{{H}}}_{2}{{O}}}-{E}_{{{{H}}}_{2}})$$

4 $$\Delta {E}_{\ast {{OH}}}=E(\ast {{OH}})-E(\ast )-({E}_{{{{H}}}_{2}{{O}}}-1/2{E}_{{{{H}}}_{2}})$$

The OER process usually summarized in four steps

5 $$\ast +{{{H}}}_{2}{{O}}\to \ast {{OH}}+{{{H}}}^{+}+{{{e}}}^{-}\cdot \qquad\qquad\qquad\Delta {G}_{{{I}}}$$

6 $$\ast {{OH}}\to \ast {{O}}+{{{H}}}^{+}+{{{e}}}^{-}\cdot \qquad\qquad\qquad\qquad\;\Delta {G}_{{{II}}}$$

7 $$\ast +{{{H}}}_{2}{{O}}\to \ast {{OOH}}+{{{H}}}^{+}+{{{e}}}^{-}\cdot \qquad\qquad\,\quad\Delta {G}_{{{III}}}$$

8 $$\ast {{OOH}}\to {{{O}}}_{2}+{{{H}}}^{+}+{{{e}}}^{-}+\ast \qquad\qquad\quad\qquad\;\Delta {G}_{{{IV}}}$$

Here * denotes adsorption active site on the substrate.

9 $$\Delta {G}_{{{I}}}=\Delta {G}_{\ast {{OH}}}$$

10 $$\Delta {G}_{{{II}}}=\Delta {G}_{\ast {{O}}}-\Delta {G}_{\ast {{OH}}}$$

11 $$\varDelta {G}_{{{III}}}=\varDelta {G}_{\ast {{OOH}}}-\varDelta {G}_{\ast {{O}}}$$

12 $$\varDelta {G}_{{{IV}}}=4.92-\Delta {G}_{\ast {{OOH}}}$$

The overpotential (*η*) is defined as below:

13 $$\eta =\,\max \{\Delta {G}_{{{I}}},\Delta {G}_{{{II}}},\Delta {G}_{{{III}}},\Delta {G}_{{{IV}}}\}-1.23\,{{eV}}$$

## Supplementary information

Supporting Information

## Data Availability

The data that support the findings of this work are available from the corresponding author upon reasonable request.
