# Supplementary material for: Engineering single-atomic ruthenium catalytic sites on defective nickel-iron layered double hydroxide for overall water splitting
Source: Nat Commun. 2021 Jul 28;12:4587. doi: 10.1038/s41467-021-24828-9 (PMC8319438; doi:10.1038/s41467-021-24828-9)
Supplement: Supplementary file 1 — Supporting Information [file 41467_2021_24828_MOESM1_ESM.pdf]

Supporting Information

Engineering single-atomic ruthenium catalytic sites on defective nickel-iron layered double hydroxide for overall water splitting

Panlong Zhai,[1,6] Mingyue Xia,[2,6] Yunzhen Wu,[1,6] Guanghui Zhang,[1] Junfeng Gao,[2] Bo Zhang,[1] Shuyan Cao,[1] Yanting Zhang,[1] Zhuwei Li,[1] Zhaozhong Fan,[1] Chen Wang,[1] Xiaomeng Zhang,[1] Jeffrey T. Miller,[5] Licheng Sun[1,3,4] and Jungang Hou[1*]

[1]State Key Laboratory of Fine Chemicals, School of Chemical Engineering, Dalian University of Technology, Dalian 116024, P. R. China.
[2]Laboratory of Materials Modification by Laser, Ion and Electron Beams (Dalian University of Technology), Ministry of Education, Dalian 116024, P. R. China.
[3]Center of Artificial Photosynthesis for Solar Fuels, School of Science, Westlake University, Hangzhou 310024, P. R. China.
[4]Department of Chemistry, School of Engineering Sciences in Chemistry, Biotechnology and Health, KTH Royal Institute of Technology, 10044 Stockholm, Sweden.
[5]Davidson School of Chemical Engineering, Purdue University, West Lafayette 47907, Indiana, United States.
[6]These authors contributed equally: Panlong Zhai, Mingyue Xia, Yunzhen Wu.

*E-mail: J.Hou (jhou@dlut.edu.cn)

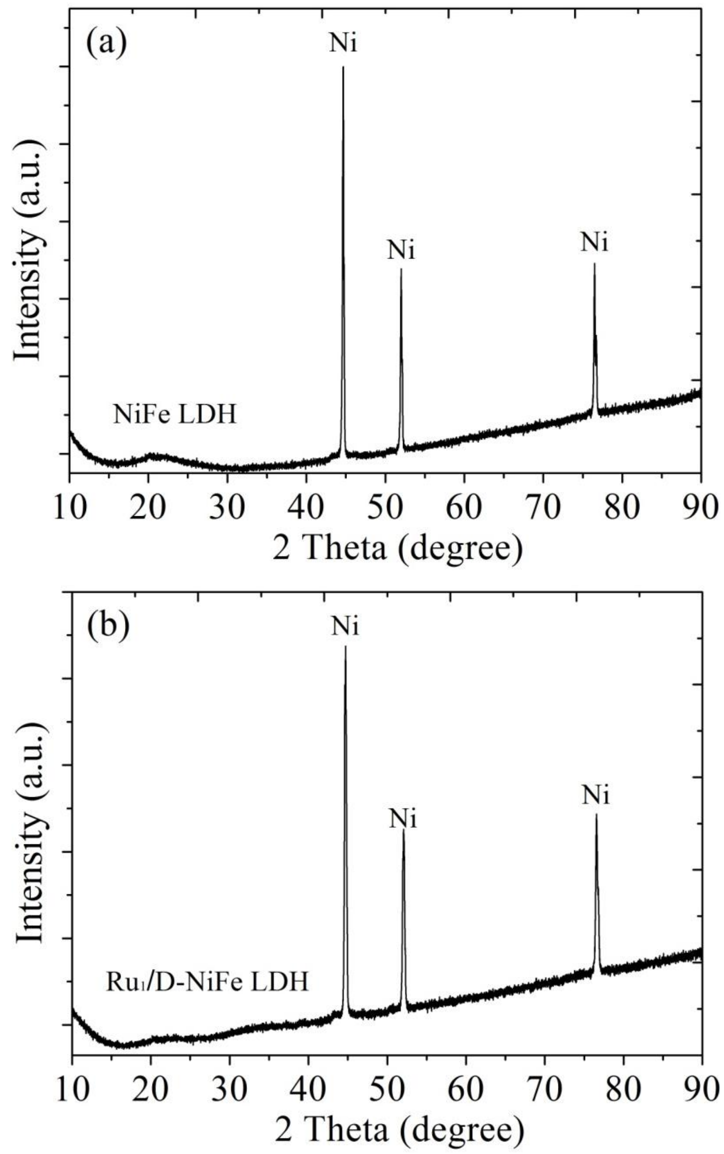

Supplementary Figure 1. XRD patterns of NiFe LDH synthesized by electrodeposition process and Ru_1/D-NiFe LDH synthesized by electrodeposition and etching process.

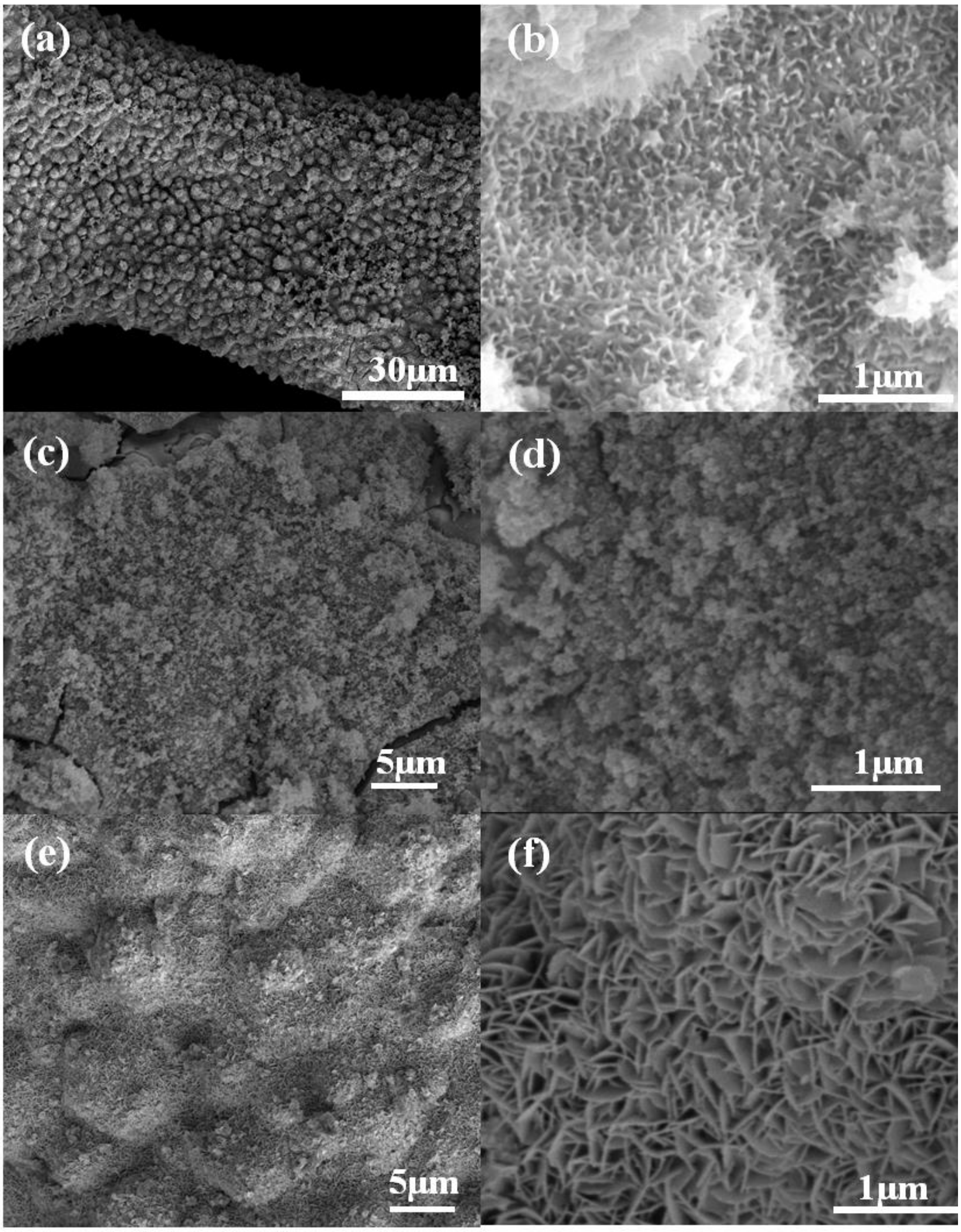

Supplementary Figure 2. SEM images of (ab) NiFe LDH and (cd) Ru_1/NiFeAl LDH synthesized by electrodeposition process, and (ef) NiFeRu LDH prepared by hydrothermal process.

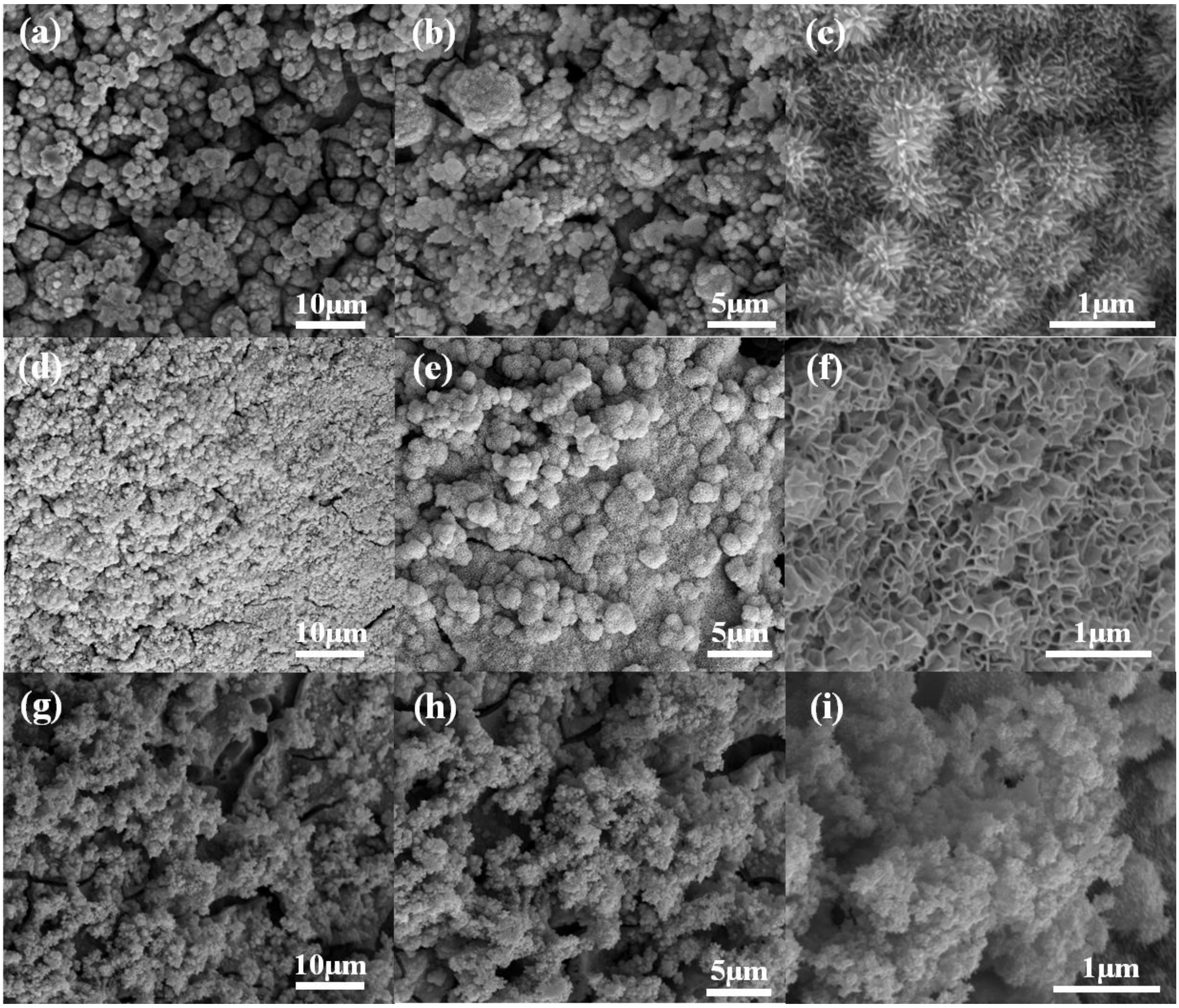

Supplementary Figure 3. SEM images of Ru_1/D-NiFe LDH by use of Ru_1/NiFeAl LDH as the precursor through etching treatment for different times, (a-c) 12 hours, (d-f) 24 hours, (g-i) 36 hours.

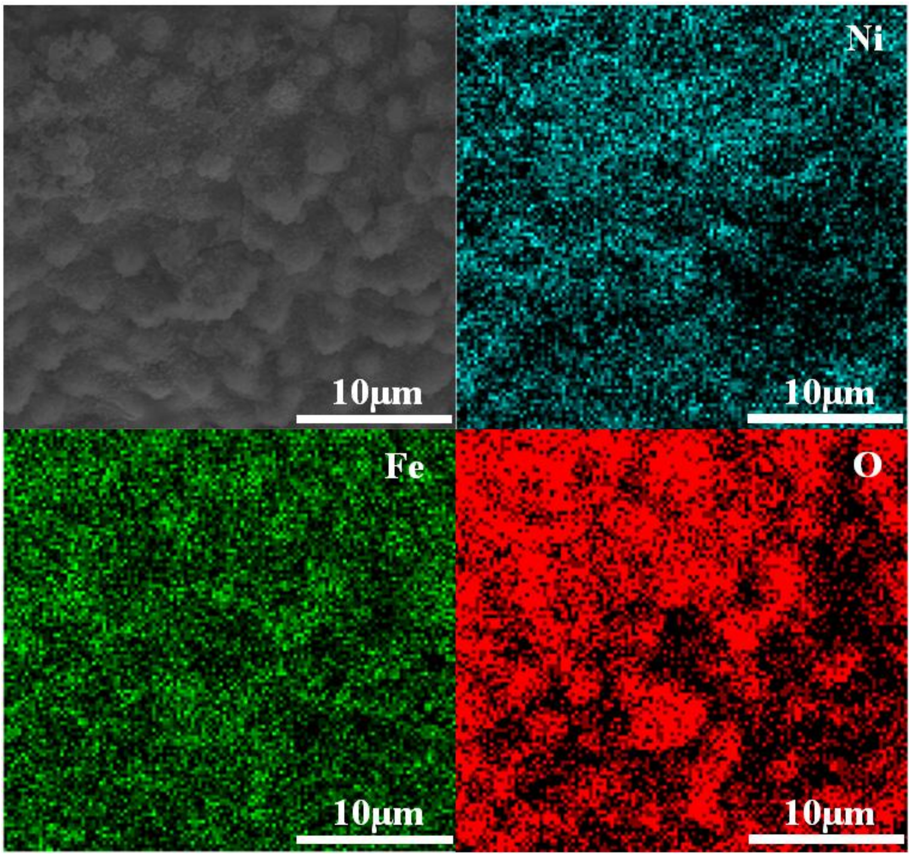

Supplementary Figure 4. SEM image and element mapping of NiFe LDH prepared by electrodeposition process.

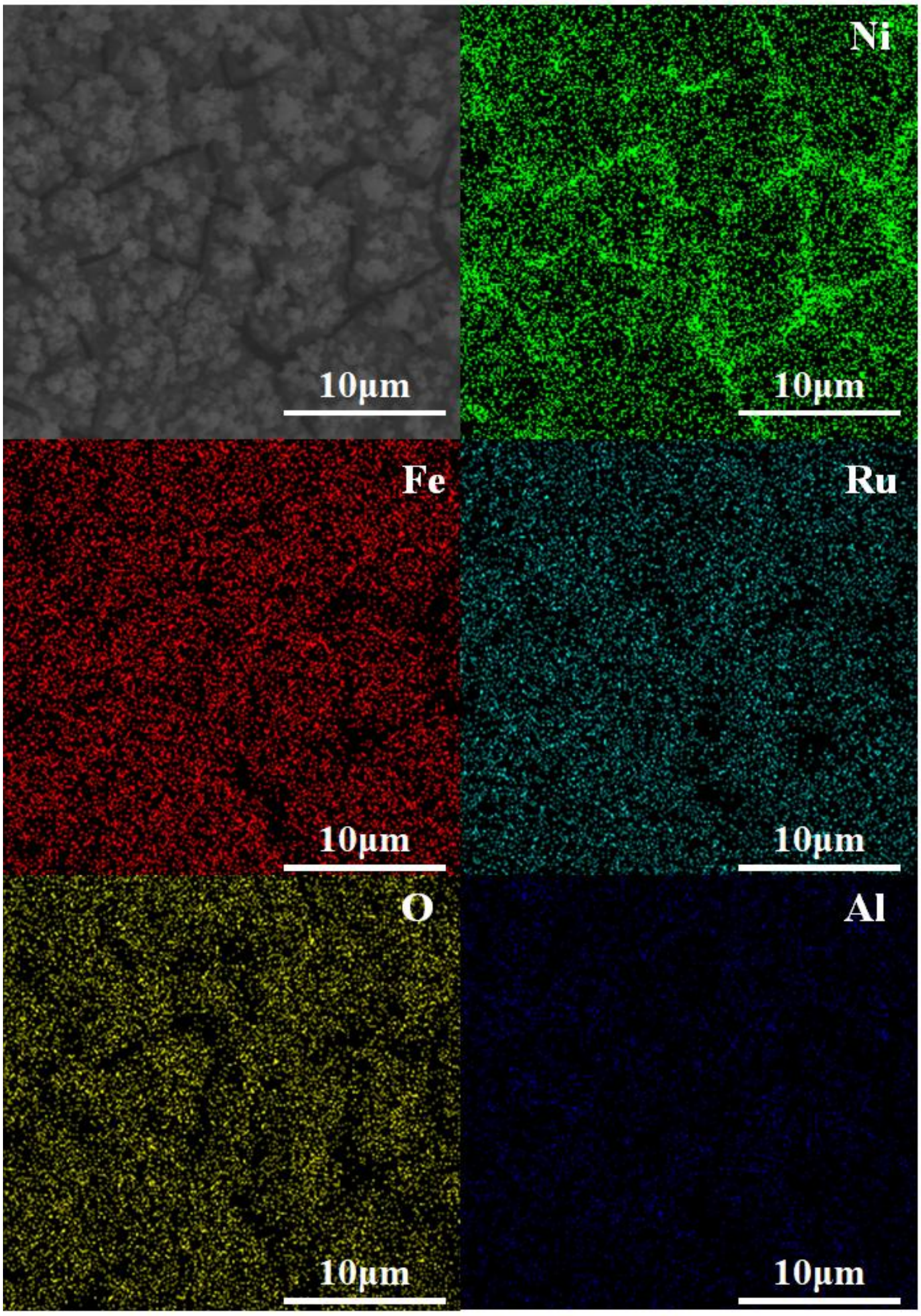

Supplementary Figure 5. SEM image and element mapping of Ru_1/NiFeAl LDH prepared by electrodeposition process.

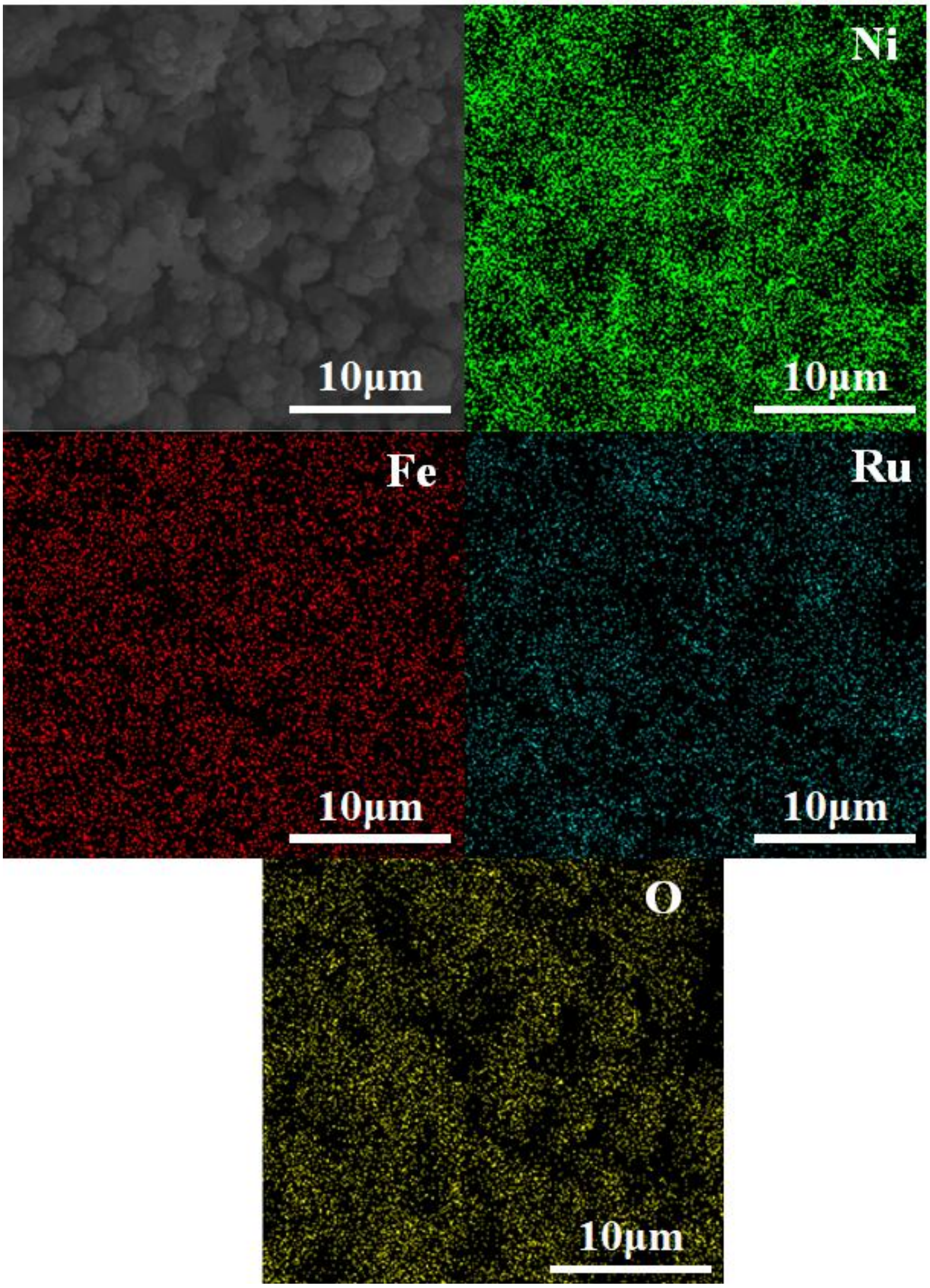

Supplementary Figure 6. SEM image and element mapping of Ru_1/D-NiFe LDH by use of Ru_1/NiFeAl LDH as the precursor through etching treatment for 12 hours.

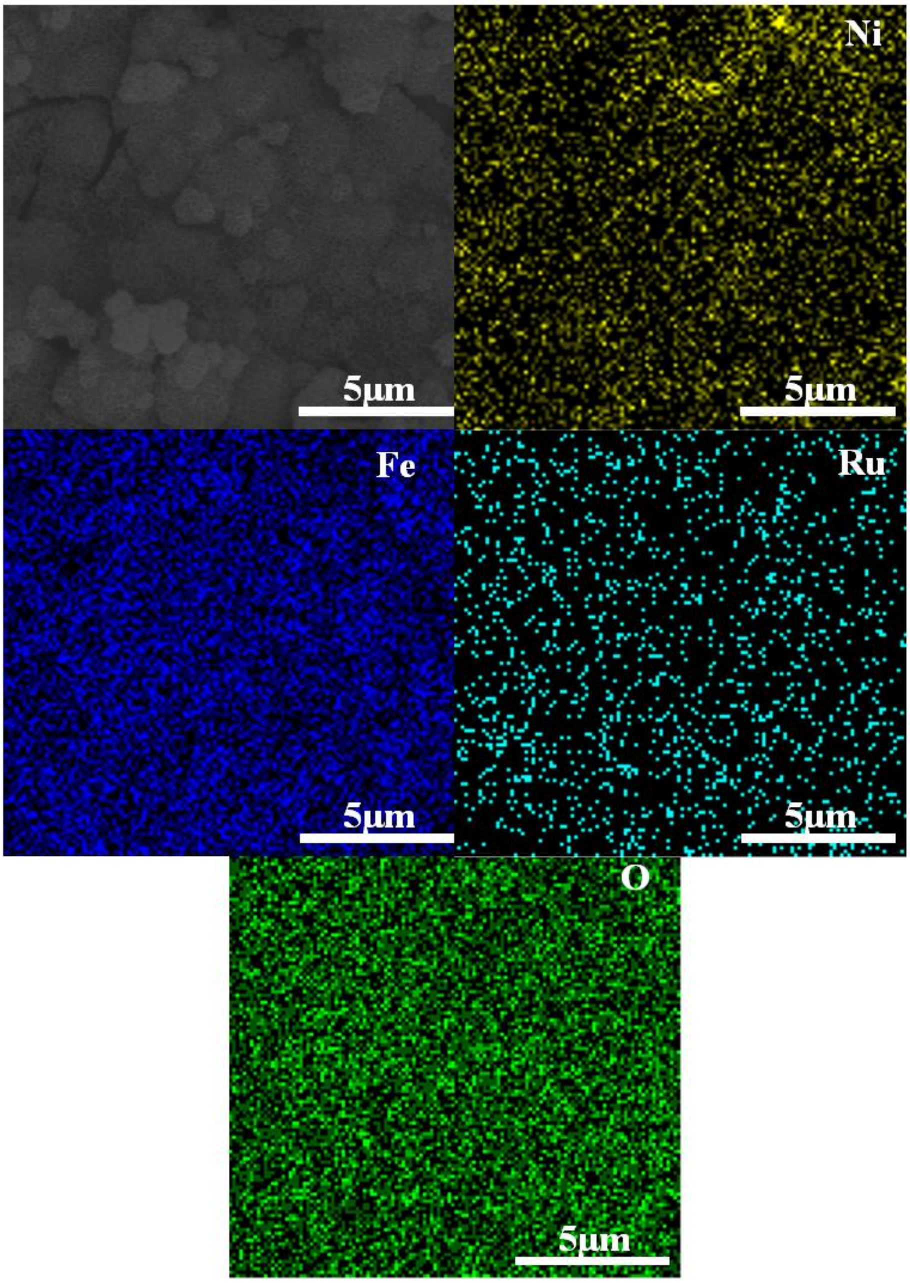

Supplementary Figure 7. SEM image and element mapping of Ru_1/D-NiFe LDH by use of Ru_1/NiFeAl LDH as the precursor through etching treatment for 24 hours.

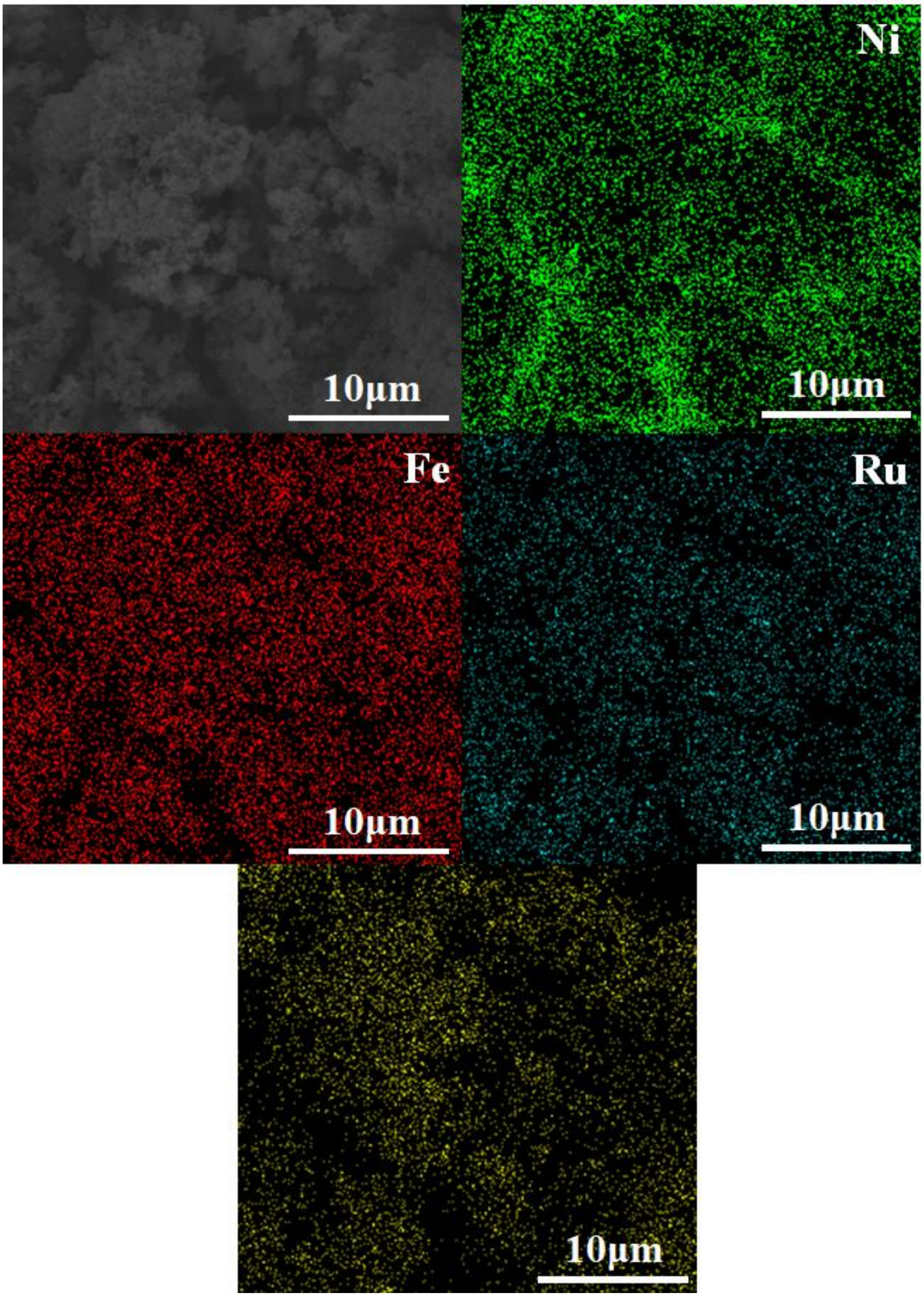

Supplementary Figure 8. SEM image and element mapping of Ru_1/D-NiFe LDH by use of Ru_1/NiFeAl LDH as the precursor through etching treatment for 36 hours.

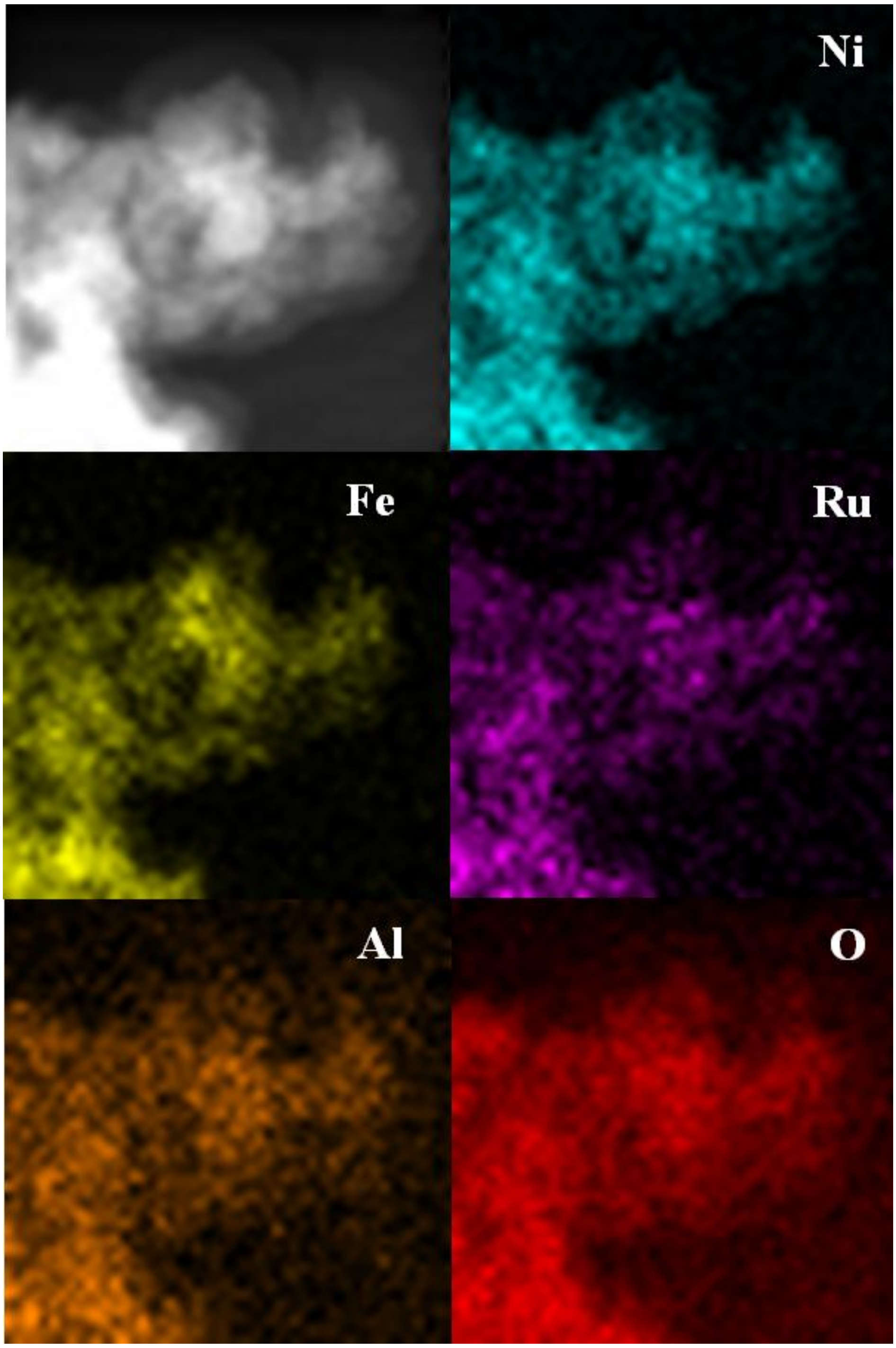

Supplementary Figure 9. High-angle annular dark-field scanning transmission electron microscopy (HAADF-STEM) image and element mapping of Ru_1/NiFeAl LDH by use of electrodeposition process.

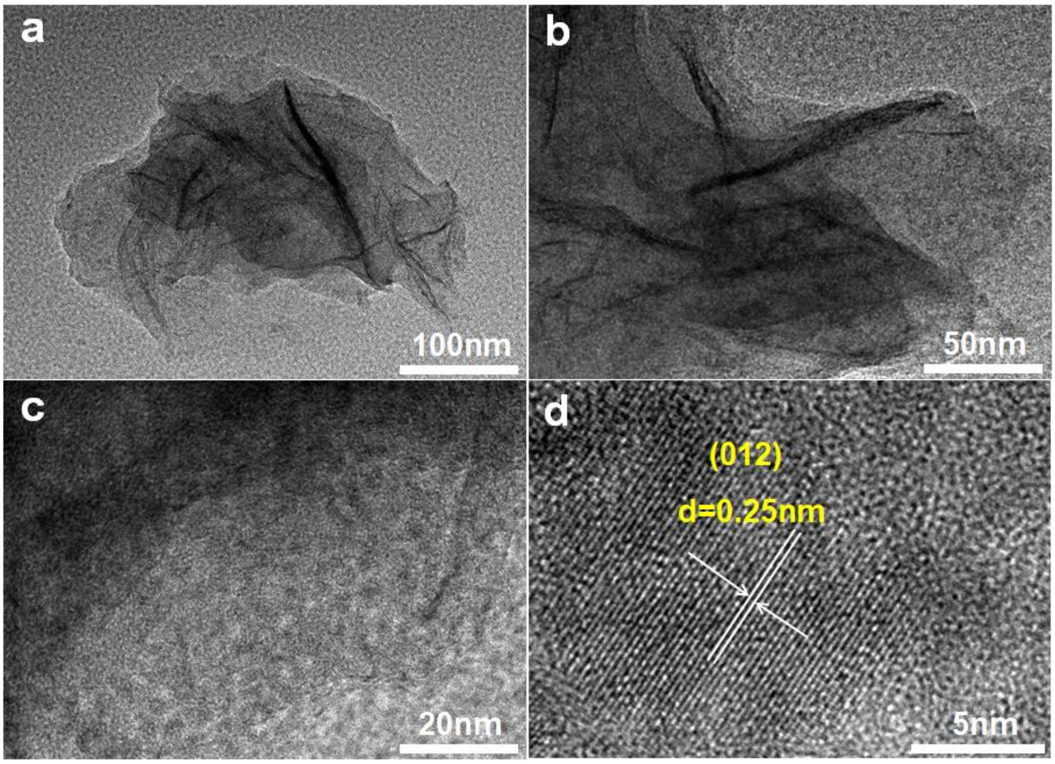

Supplementary Figure 10. (a-c) Transmission electron microscopy images and (d) high-resolution transmission electron microscopy images of Ru doped NiFe LDH.

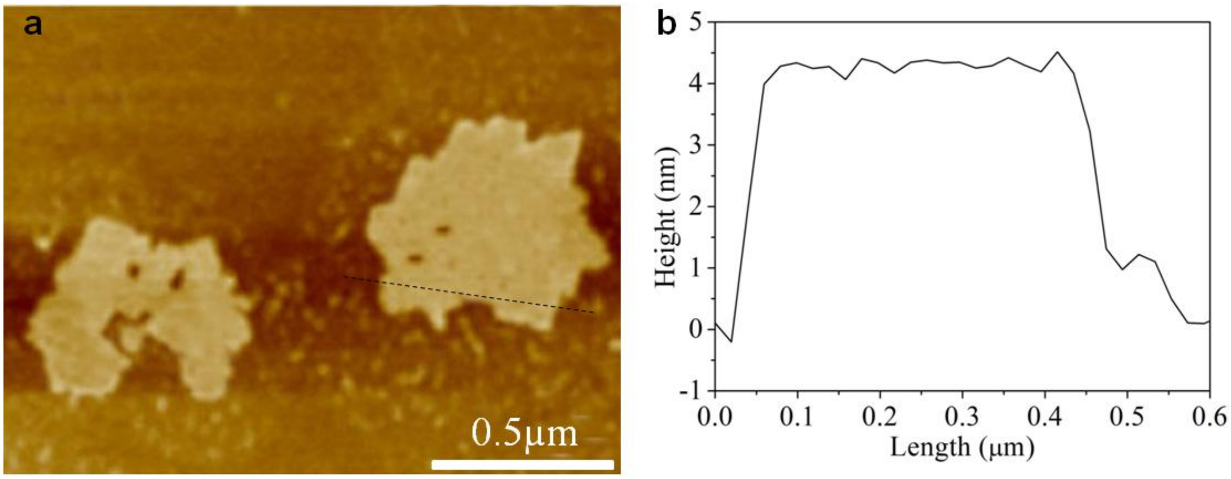

Supplementary Figure 11. (a) AFM image and (b) corresponding height profile of Ru_1/D-NiFe LDH nanosheets.

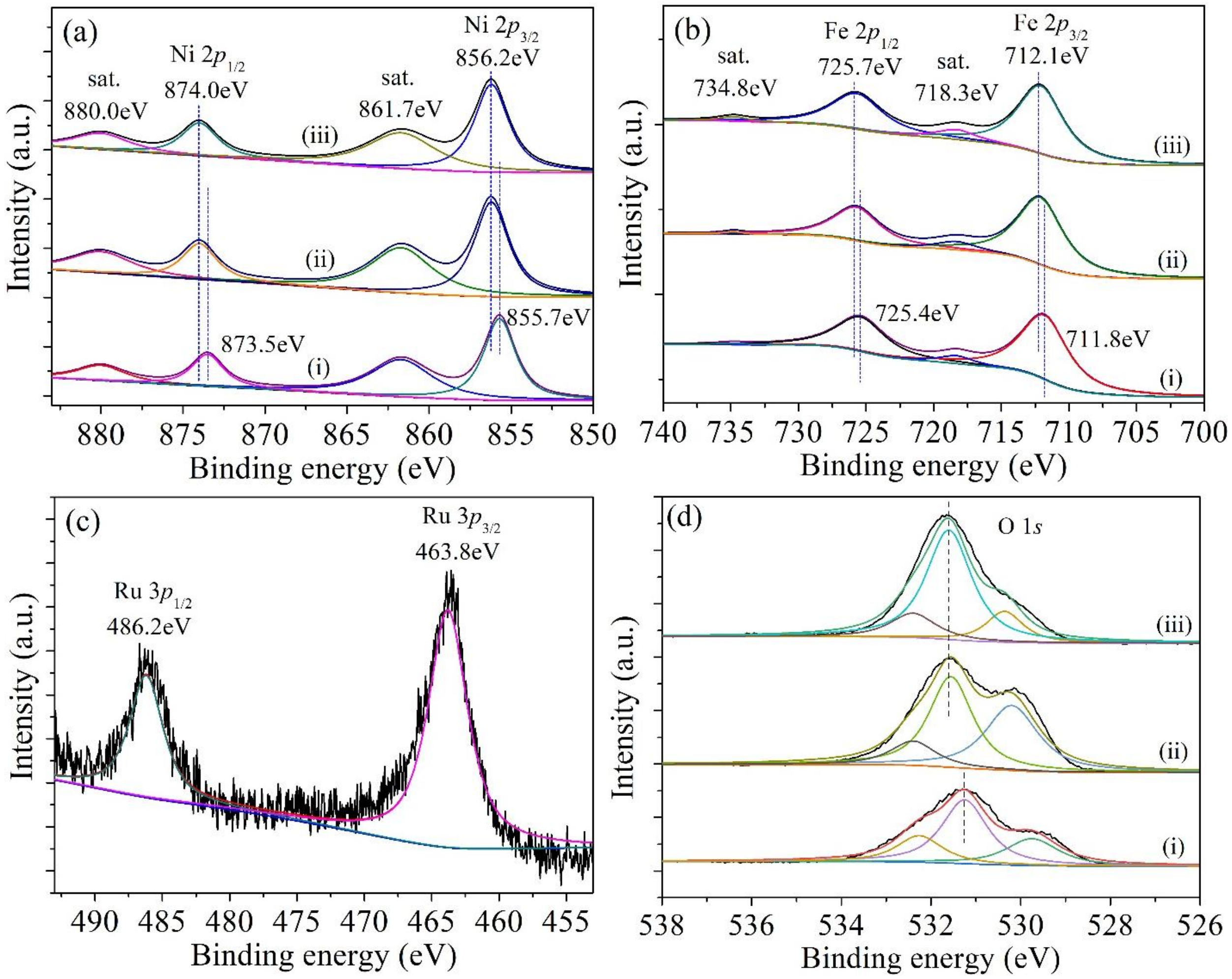

Supplementary Figure 12. (a) Ni 2*p*, (b) Fe 2*p* and (d) O 1*s* of (i) NiFe LDH, (ii) Ru_1/NiFe LDH and (iii) Ru_1/D-NiFe LDH, (c) Ru 3p of Ru_1/D-NiFe LDH.

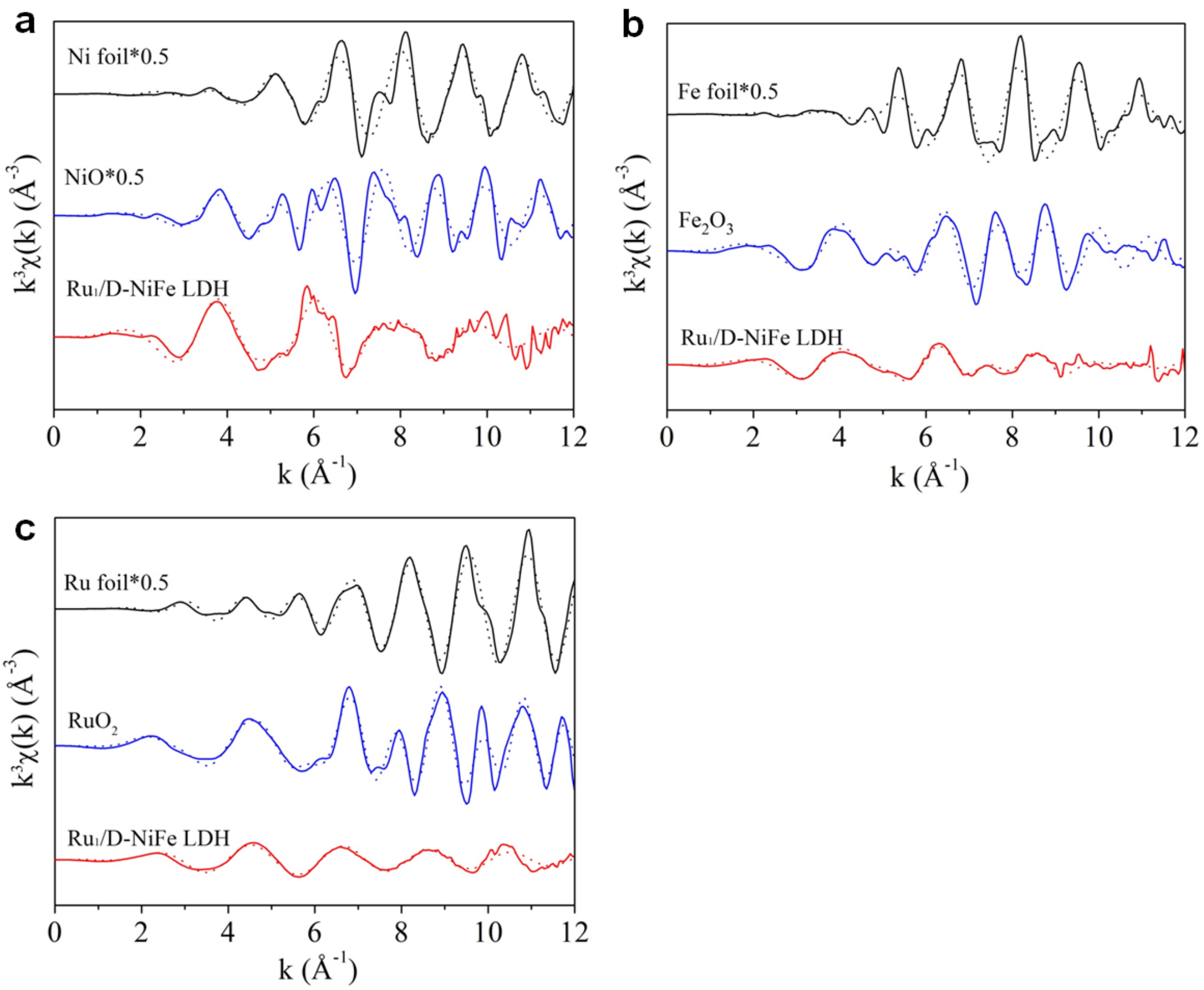

Supplementary Figure 13. (a) Ni K-edge EXAFS spectra in k space for Ru_1/D-NiFe LDH, Ni foil and NiO, (b) Fe K-edge EXAFS spectra in k space for Ru_1/D-NiFe LDH, Fe foil and Fe_2O_3, (c) Ru K-edge EXAFS spectra in k space for Ru_1/D-NiFe LDH, Ru foil and RuO_2.

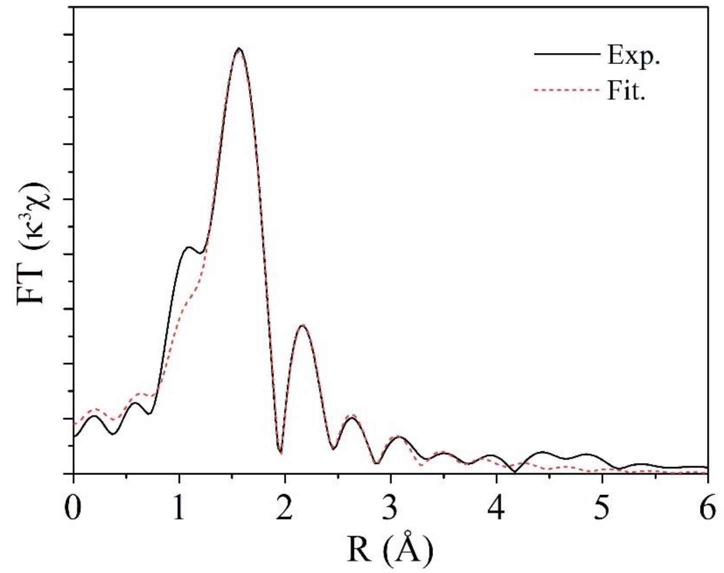

Supplementary Figure 14. Corresponding model-based fittings of Ru EXAFS for Ru_1/D-NiFe LDH and simulated EXAFS spectra.

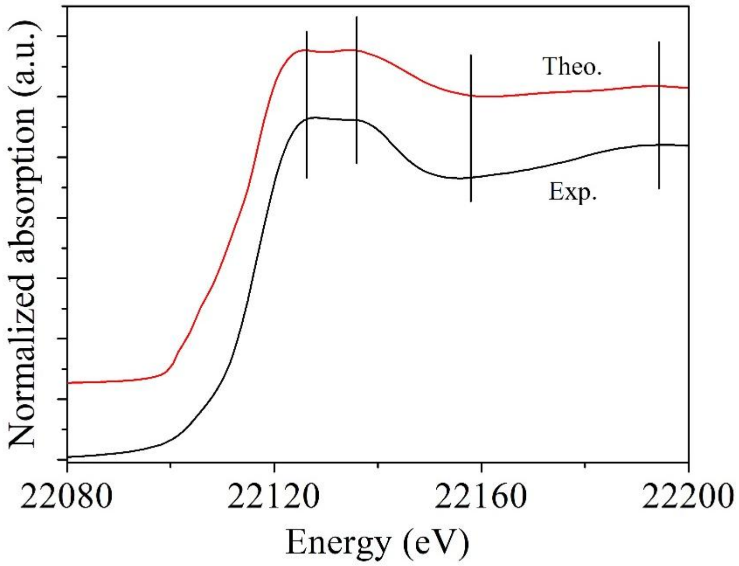

Supplementary Figure 15. The experimental XANES curves and calculated XANES data of Ru_1/D-NiFe LDH.

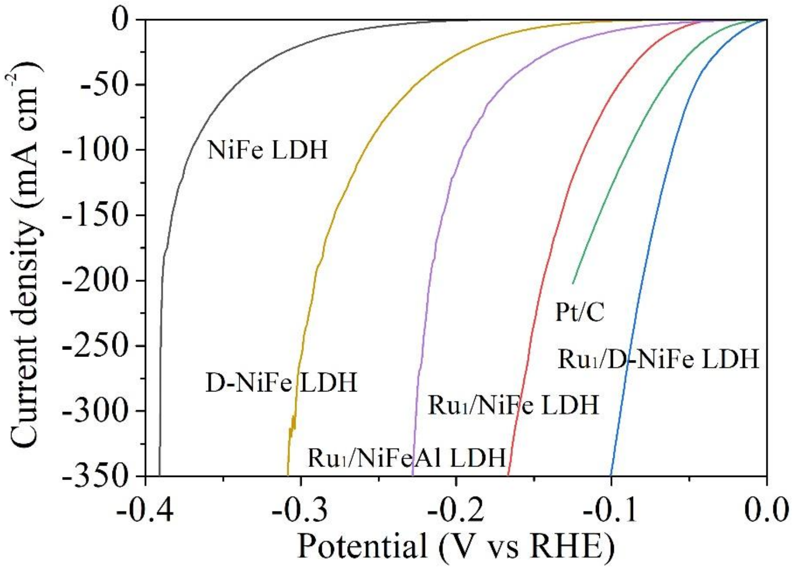

Supplementary Figure 16. HER performance of Pt/C, NiFe LDH, Ru_1/NiFe LDH, Ru_1/D-NiFe LDH, Ru_1/NiFeAl LDH and D-NiFe LDH.

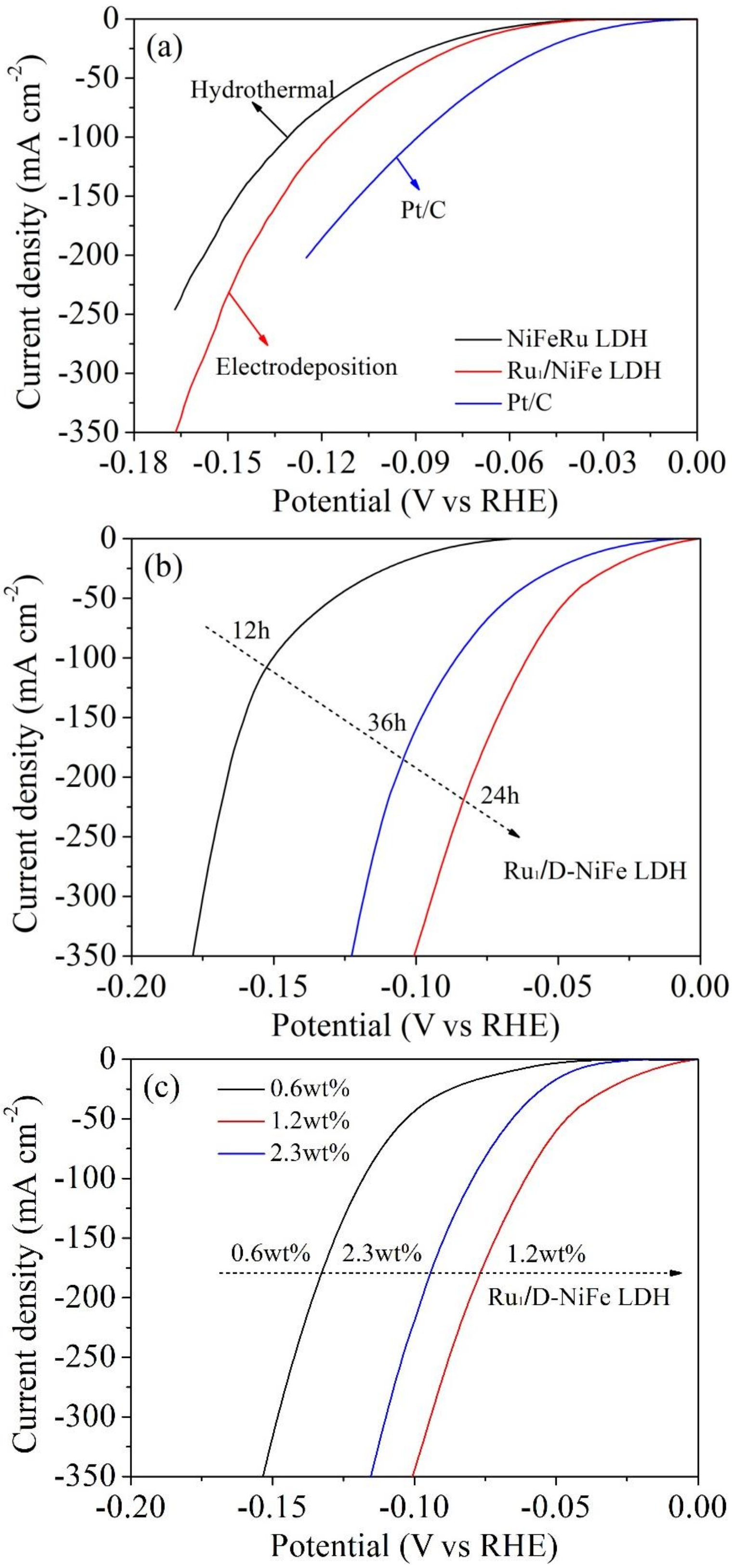

Supplementary Figure 17. HER performance of (a) NiFeRu LDH and Ru_1/NiFe LDH, (b) Ru_1/D-NiFe LDH with various etching times and (c) Ru_1/D-NiFe LDH with various contents of Ru.

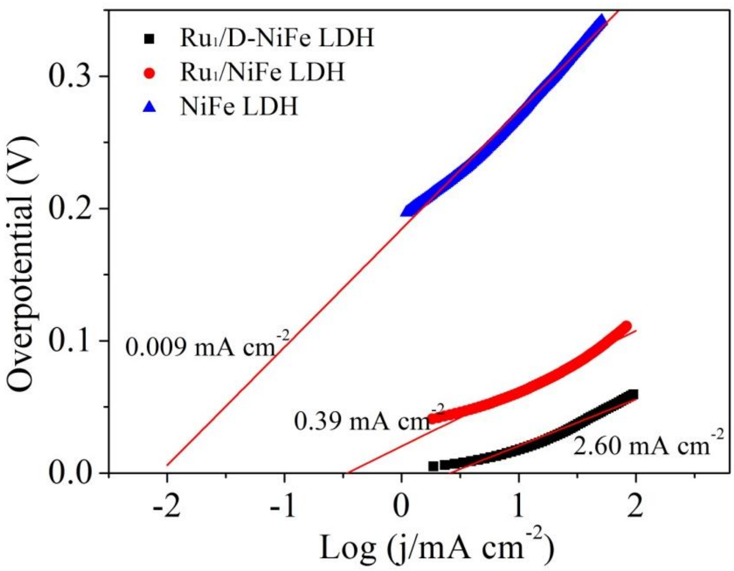

Supplementary Figure 18. The exchange current densities of the Ru_1/D-NiFe LDH, Ru_1/NiFe LDH and NiFe LDH electrocatalysts.

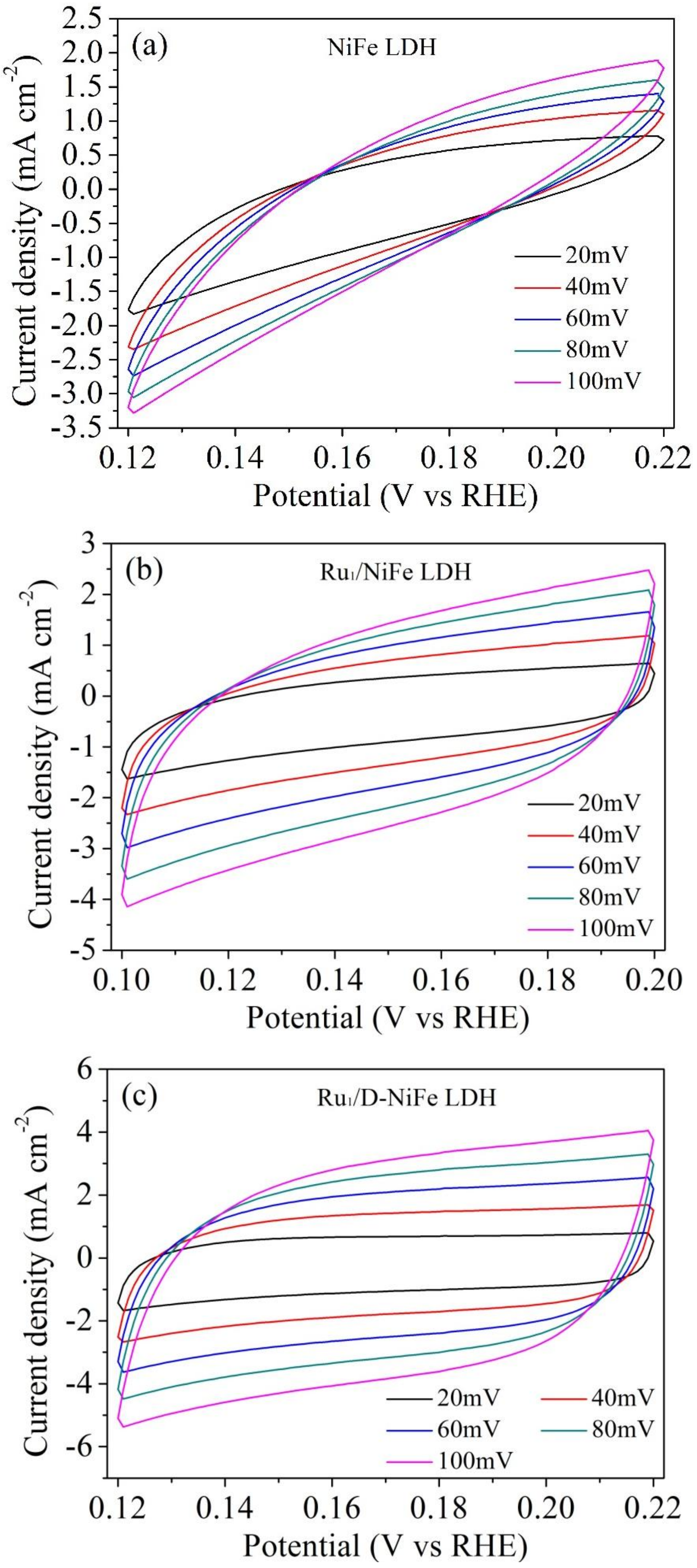

Supplementary Figure 19. Cyclic voltammograms of (a) NiFe LDH, (b) Ru_1/NiFe LDH and (c) Ru_1/D-NiFe LDH at various scan rates for HER.

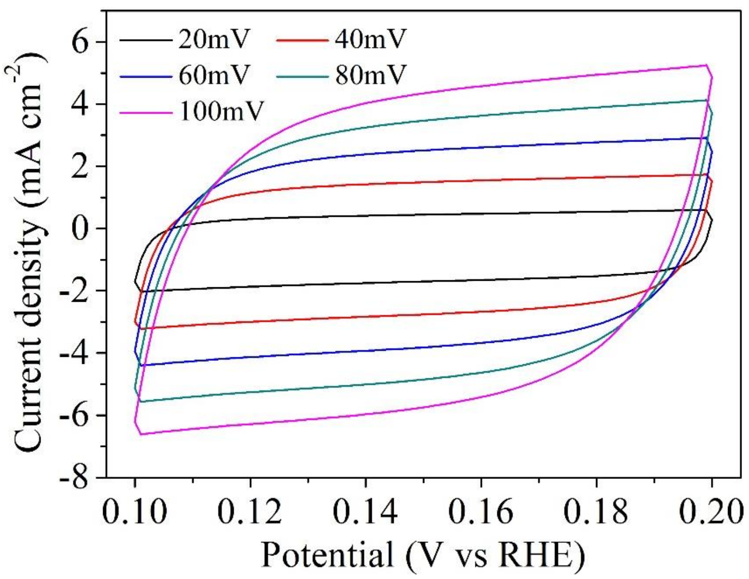

Supplementary Figure 20. Cyclic voltammograms of 20wt% Pt/C at various scan rates for HER.

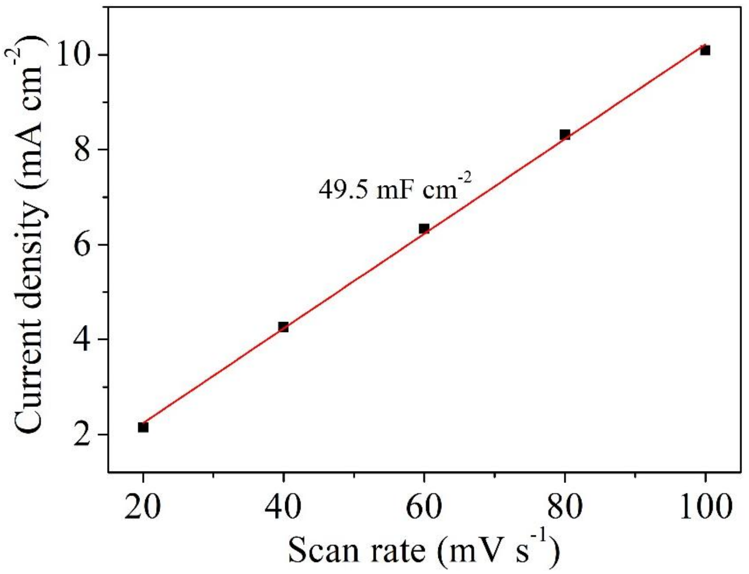

Supplementary Figure 21. Double-layer capacitances (C_{dl}) calculated by the differences in current density as a linear function of scan rates of 20wt% Pt/C.

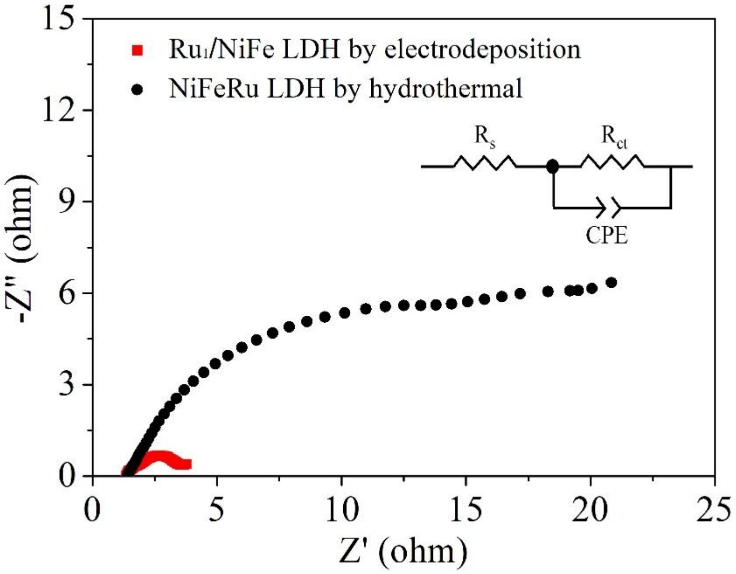

Supplementary Figure 22. Electrochemical impedance spectroscopies of (a) NiFeRu LDH and Ru_1/NiFe LDH.

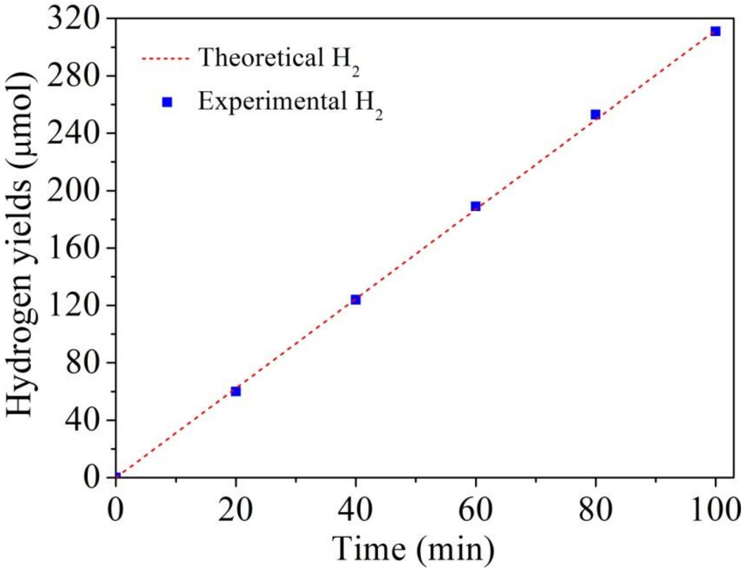

Supplementary Figure 23. The amount of gas theoretically calculated and experimentally measured vs. time for HER by use of Ru_1/D-NiFe LDH.

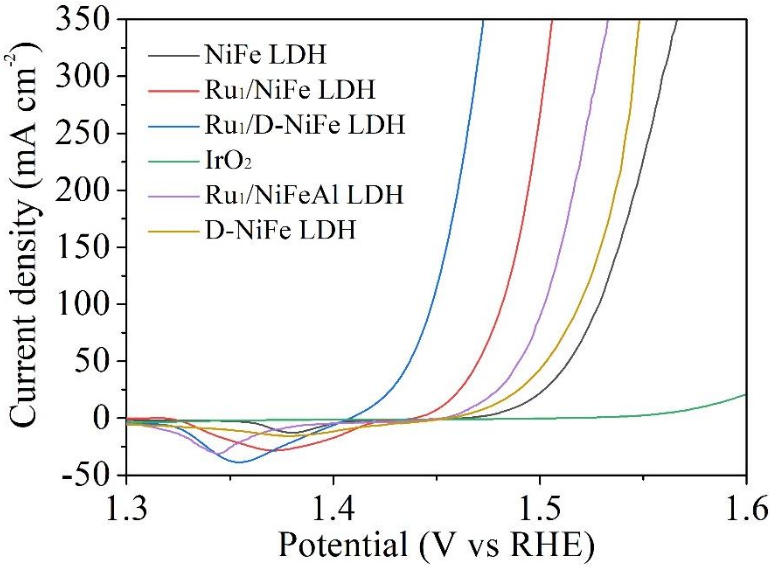

Supplementary Figure 24. OER performance of IrO_2, NiFe LDH, Ru_1/NiFe LDH, Ru_1/D-NiFe LDH, Ru_1/NiFeAl LDH and D-NiFe LDH.

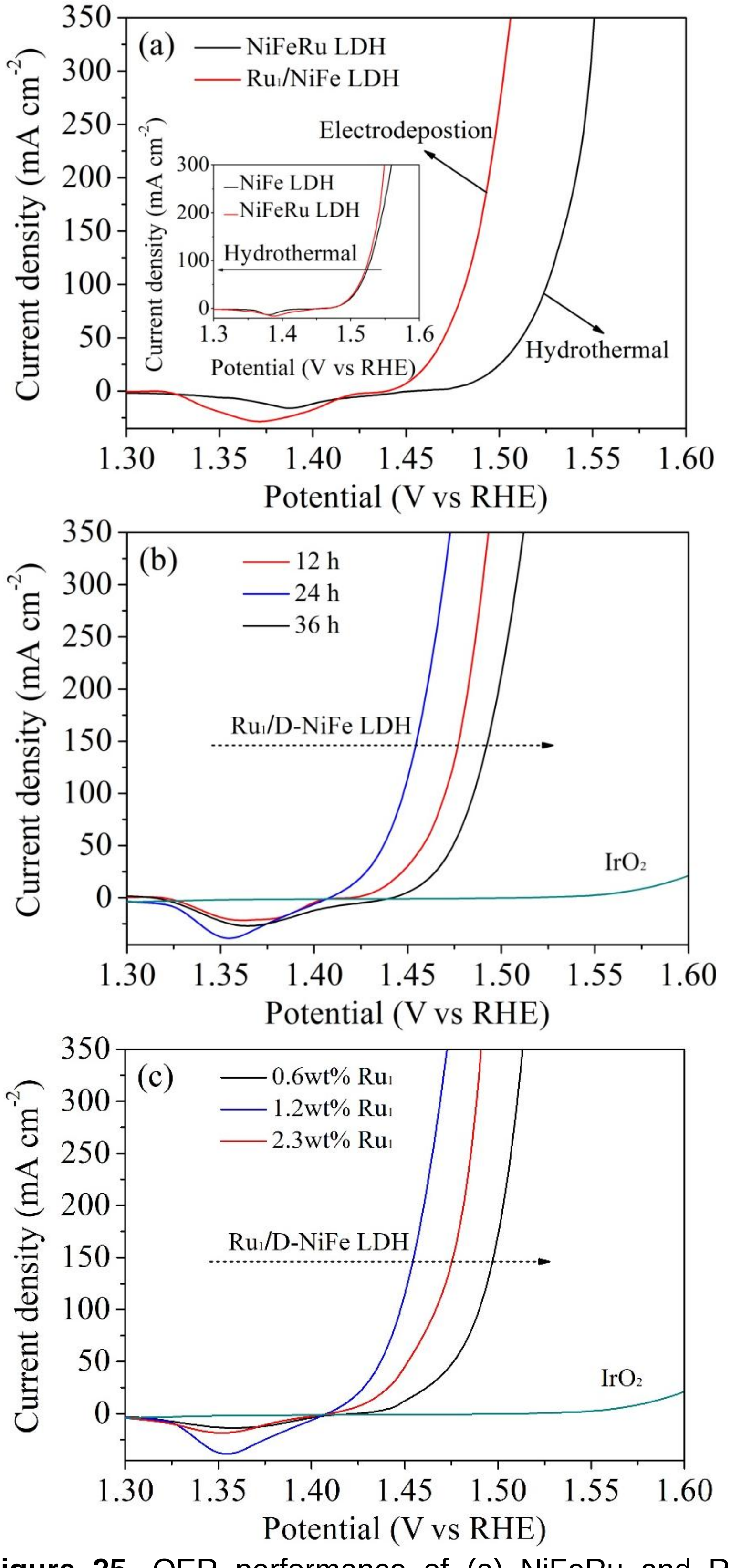

Supplementary Figure 25. OER performance of (a) NiFeRu and Ru_1/NiFe LDH, (b) Ru_1/D-NiFe LDH with various etching times and (c) Ru_1/D-NiFe LDH with various contents of Ru single atoms.

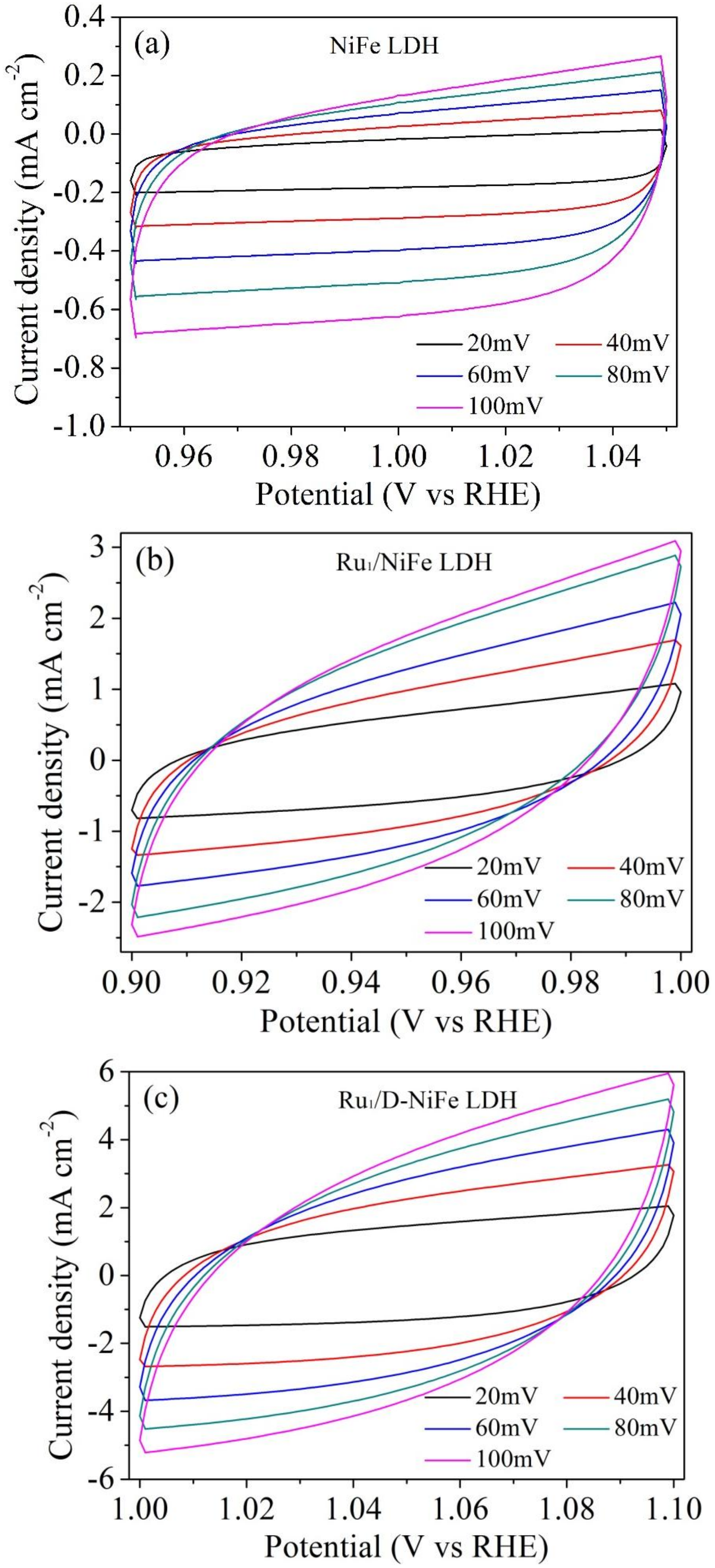

Supplementary Figure 26. Cyclic voltammograms of (a) NiFe LDH, (b) Ru_1/NiFe LDH and (c) Ru_1/D-NiFe LDH at various scan rates for OER.

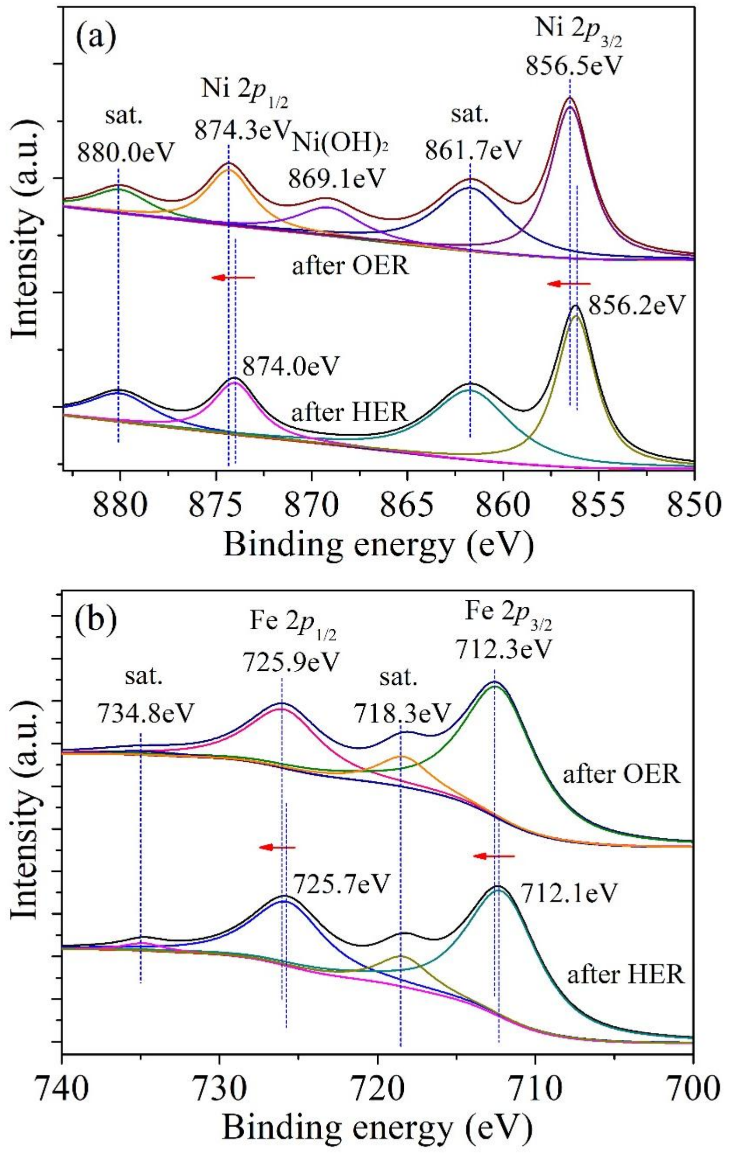

Supplementary Figure 27. (a) Ni 2*p* and (b) Fe 2*p* XPS spectra of Ru_1/D-NiFe LDH after long-term HER and OER electrocatalysis.

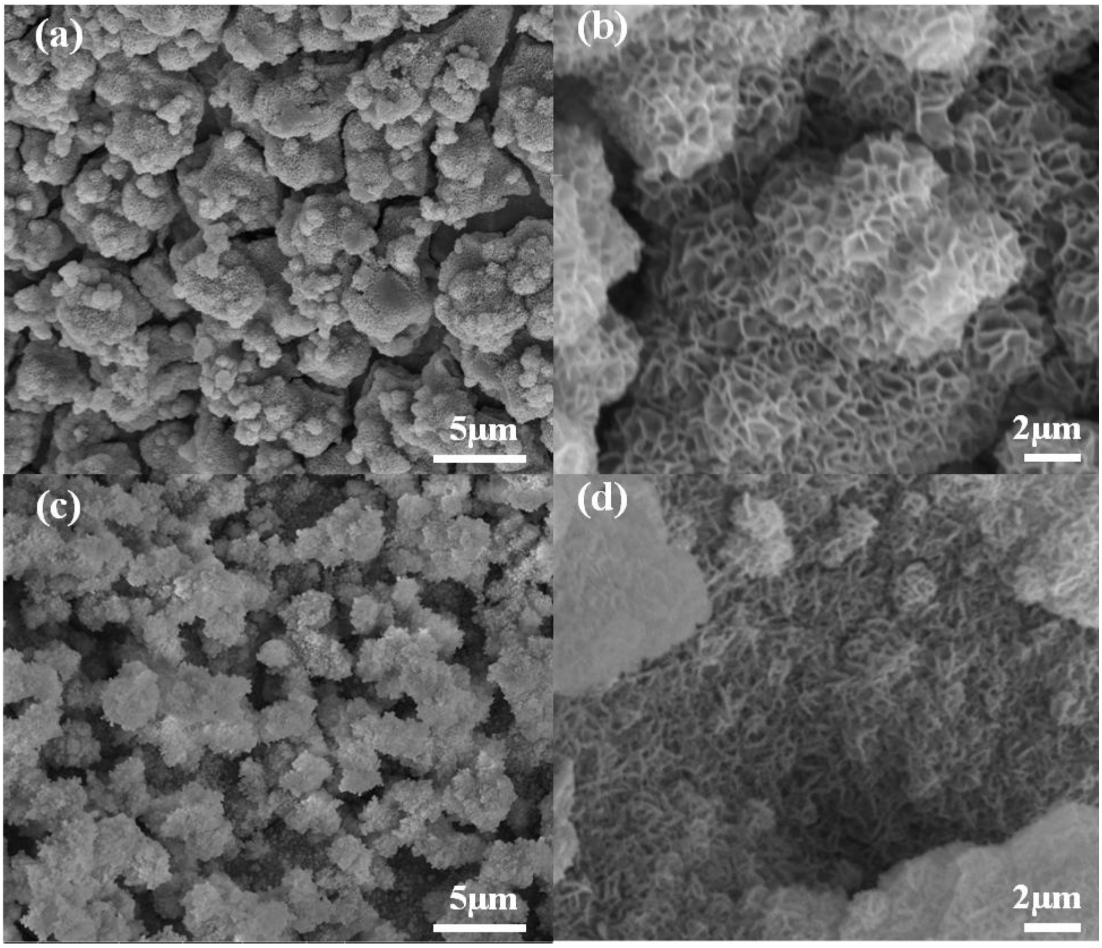

Supplementary Figure 28. SEM images of Ru_1/D-NiFe LDH for (ab) HER and (cd) OER after long-term electrocatalysis.

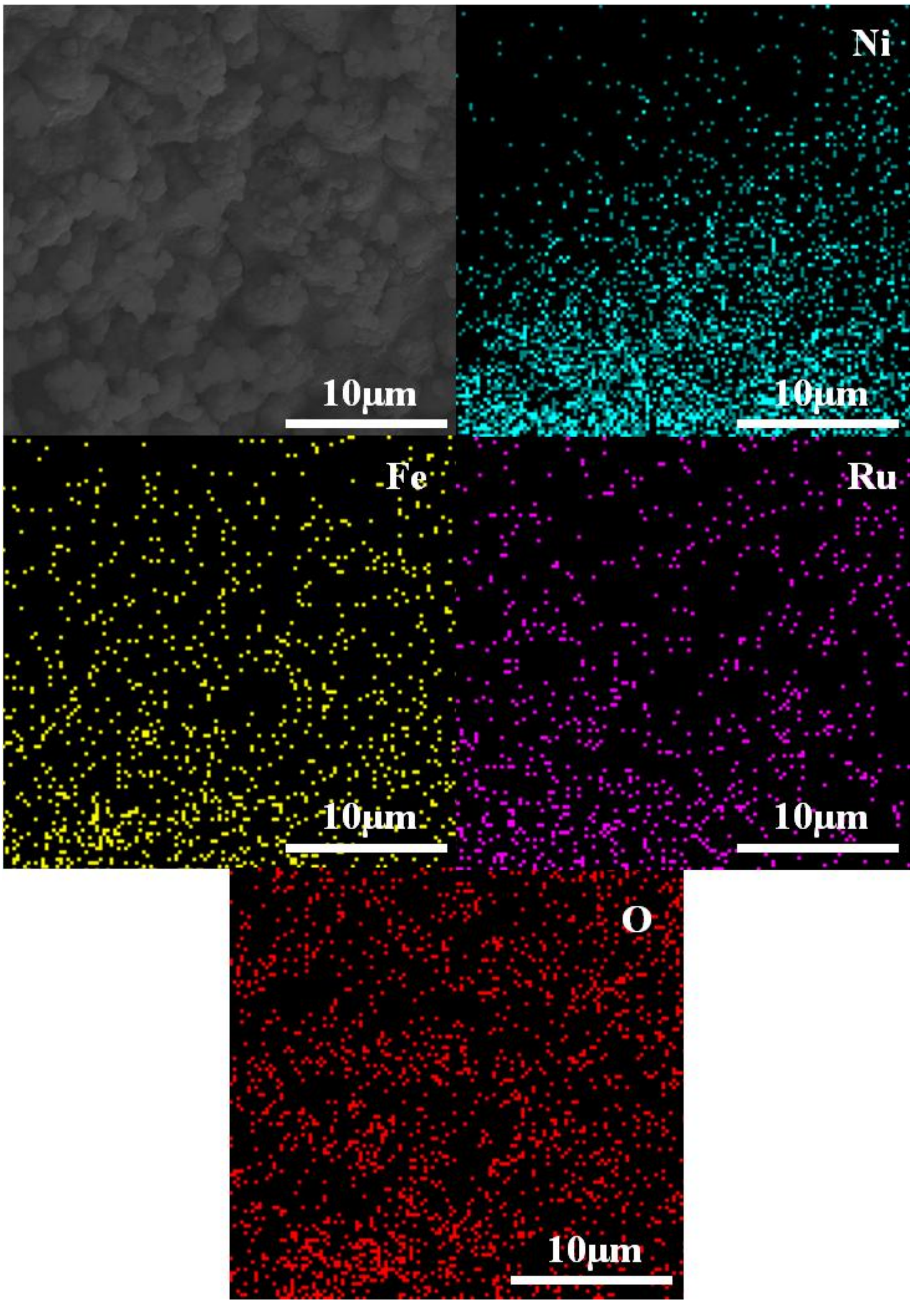

Supplementary Figure 29. SEM image and element mapping images of Ru_1/D-NiFe LDH after HER.

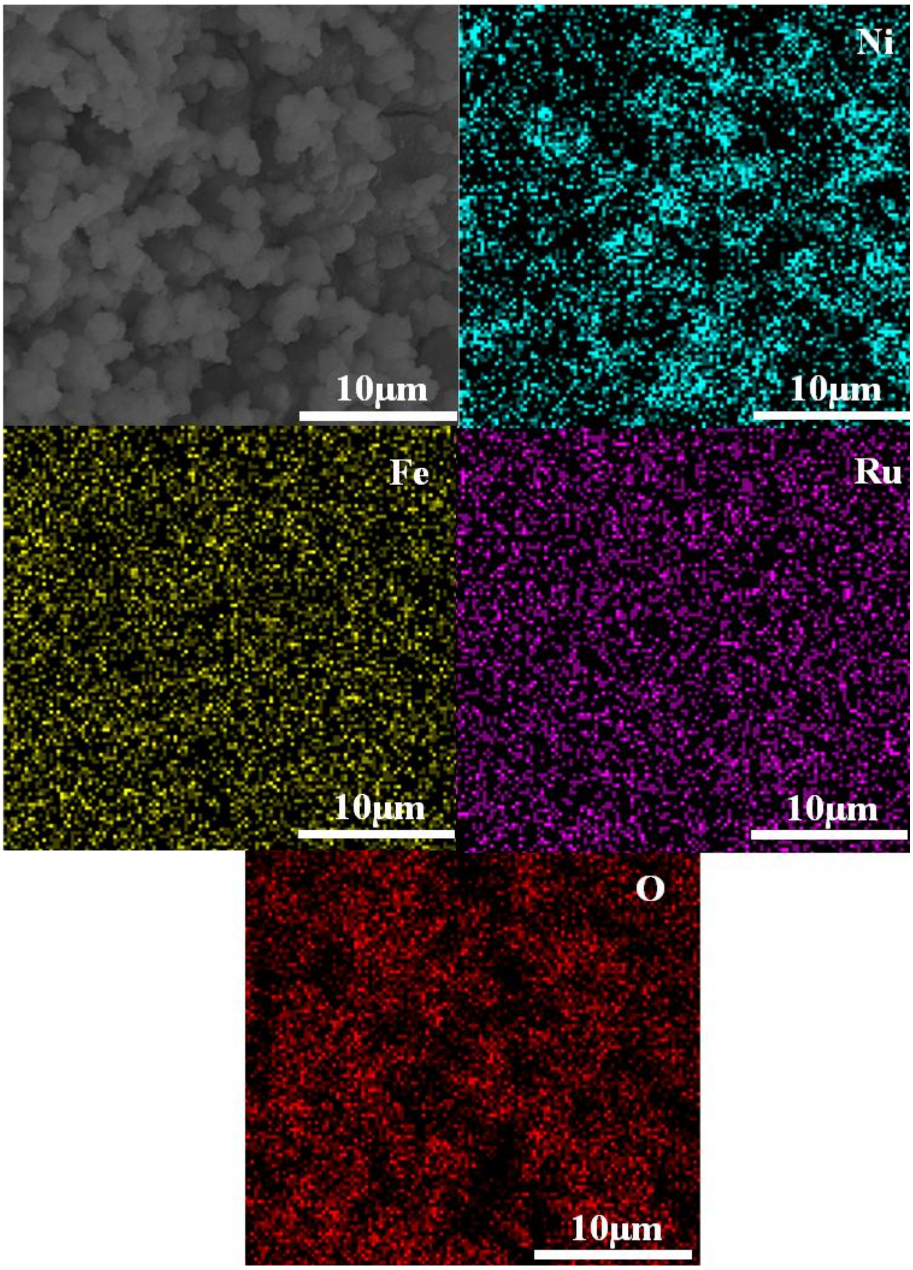

Supplementary Figure 30. SEM image and element mapping images of Ru_1/D-NiFe LDH after OER.

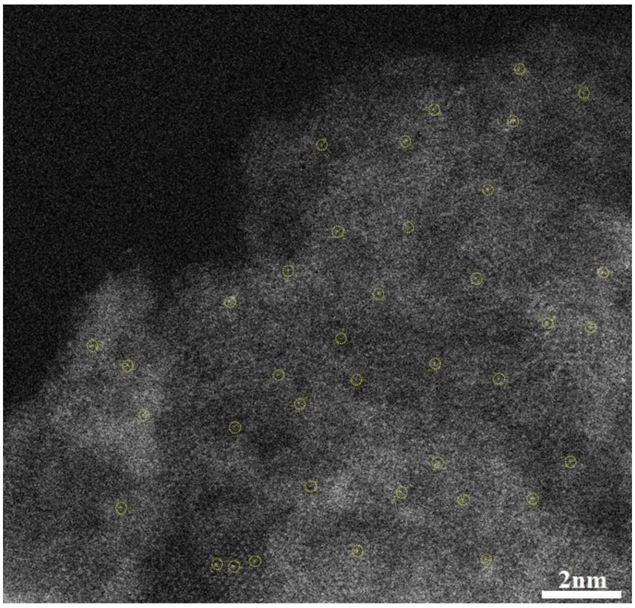

Supplementary Figure 31. The image of Ru_1/D-NiFe LDH by aberration-corrected transmission electron microscope after long-term electrocatalysis.

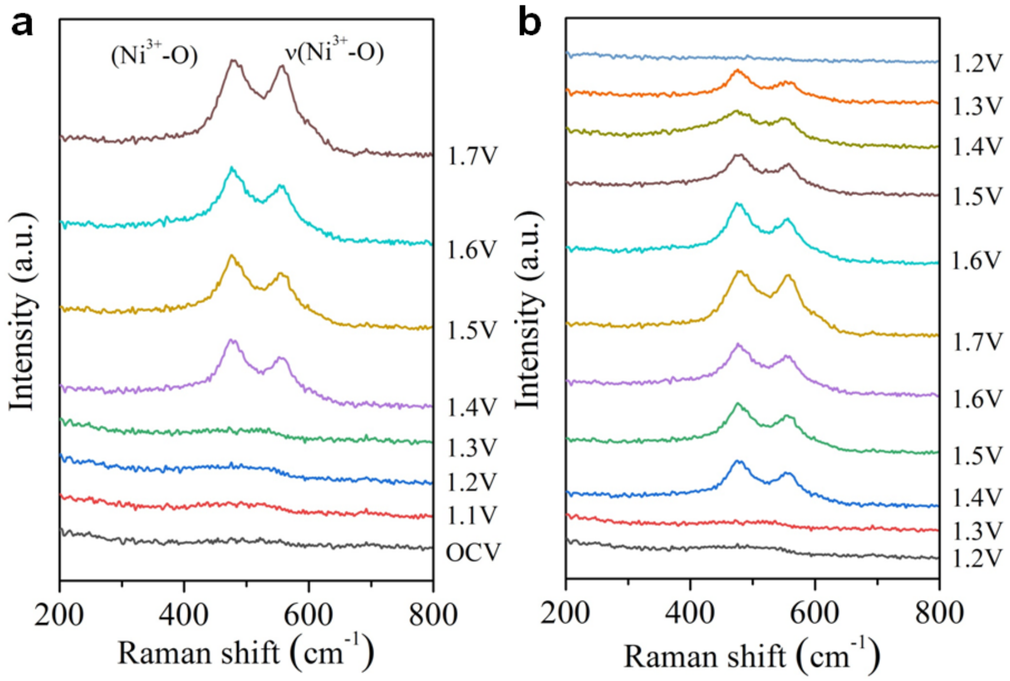

Supplementary Figure 32. In-situ Raman spectra of Ru_1/D-NiFe LDH (a) from open circuit voltage to 1.7 V vs. RHE in 1 M KOH electrolyte, (b) from 1.2 V to 1.7 V and reverse to 1.2 V vs. RHE.

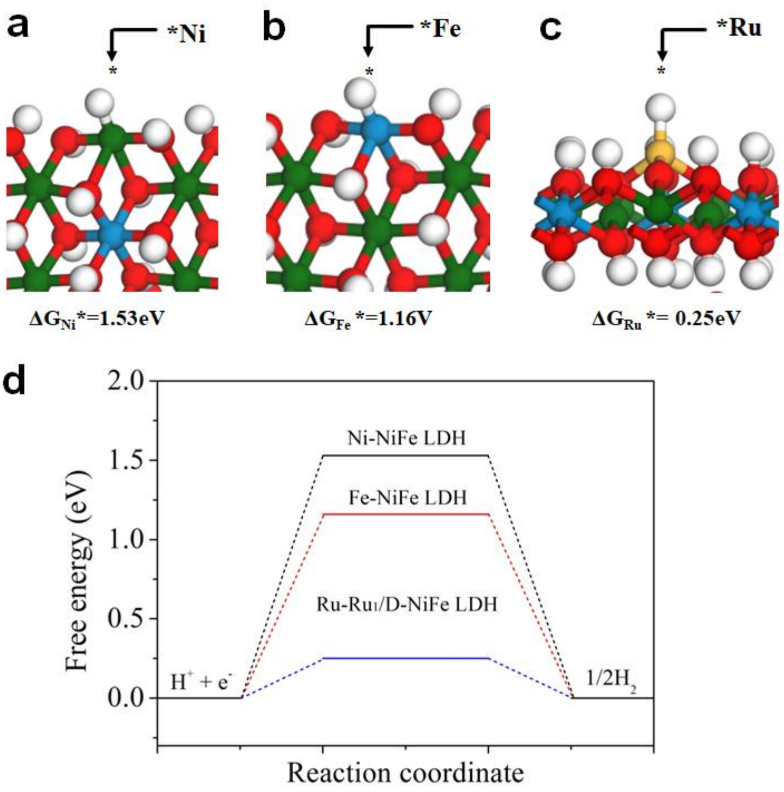

Supplementary Figure 33. Chemisorption models of H at the (a) Ni, (b) Fe site in NiFe LDH and (c) Ru site on Ru_1/D-NiFe LDH. (d) Free energy diagram of HER process on different active sites for NiFe LDH and Ru_1/D-NiFe LDH.

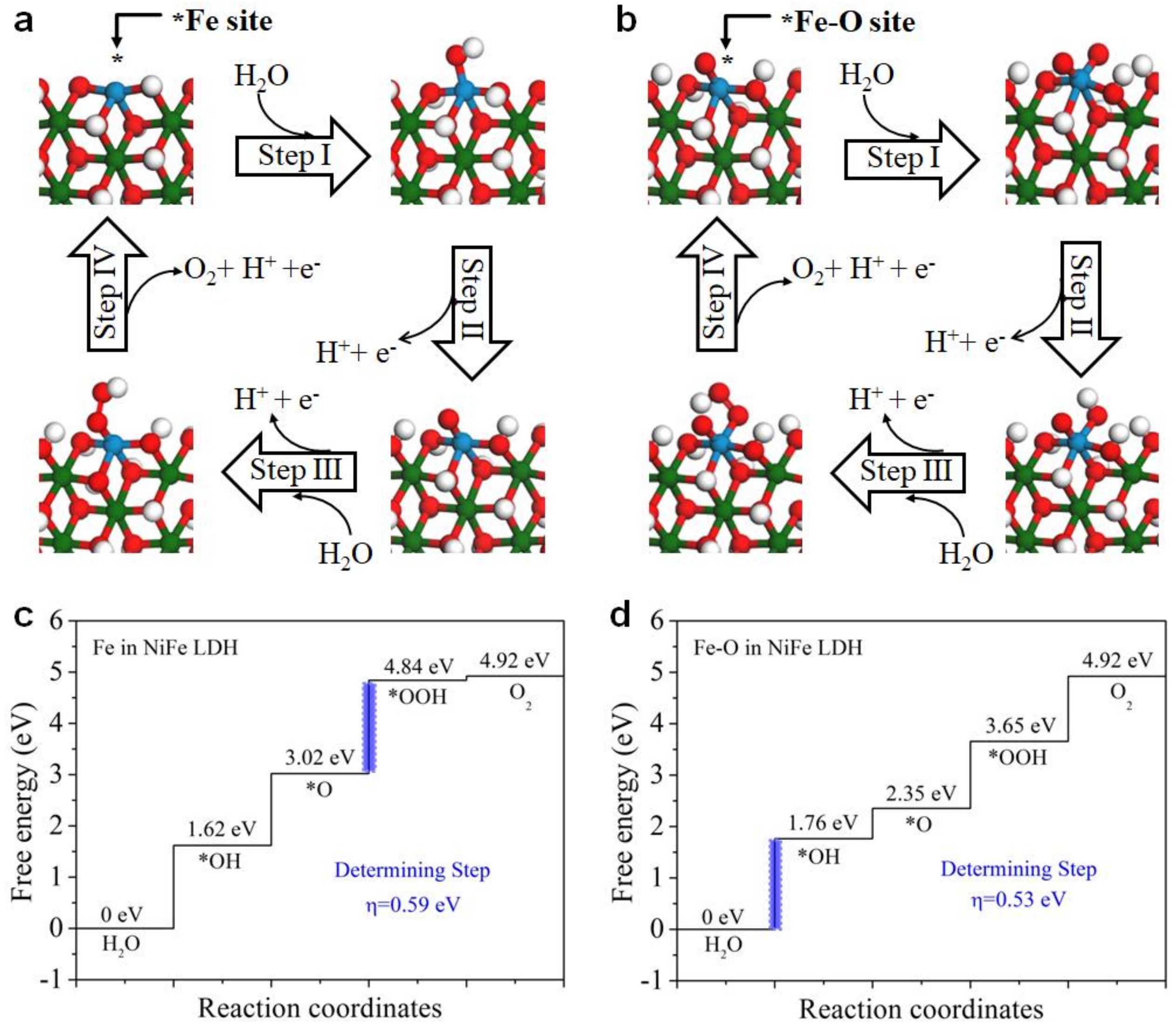

Supplementary Figure 34. Schematic illustration of the proposed OER mechanism for (a) Fe and (b) Fe–O sites in NiFe LDH. Gibbs free energy diagram for (c) Fe and (d) Fe–O sites in NiFe LDH. The lavender box step is the rate determining step.

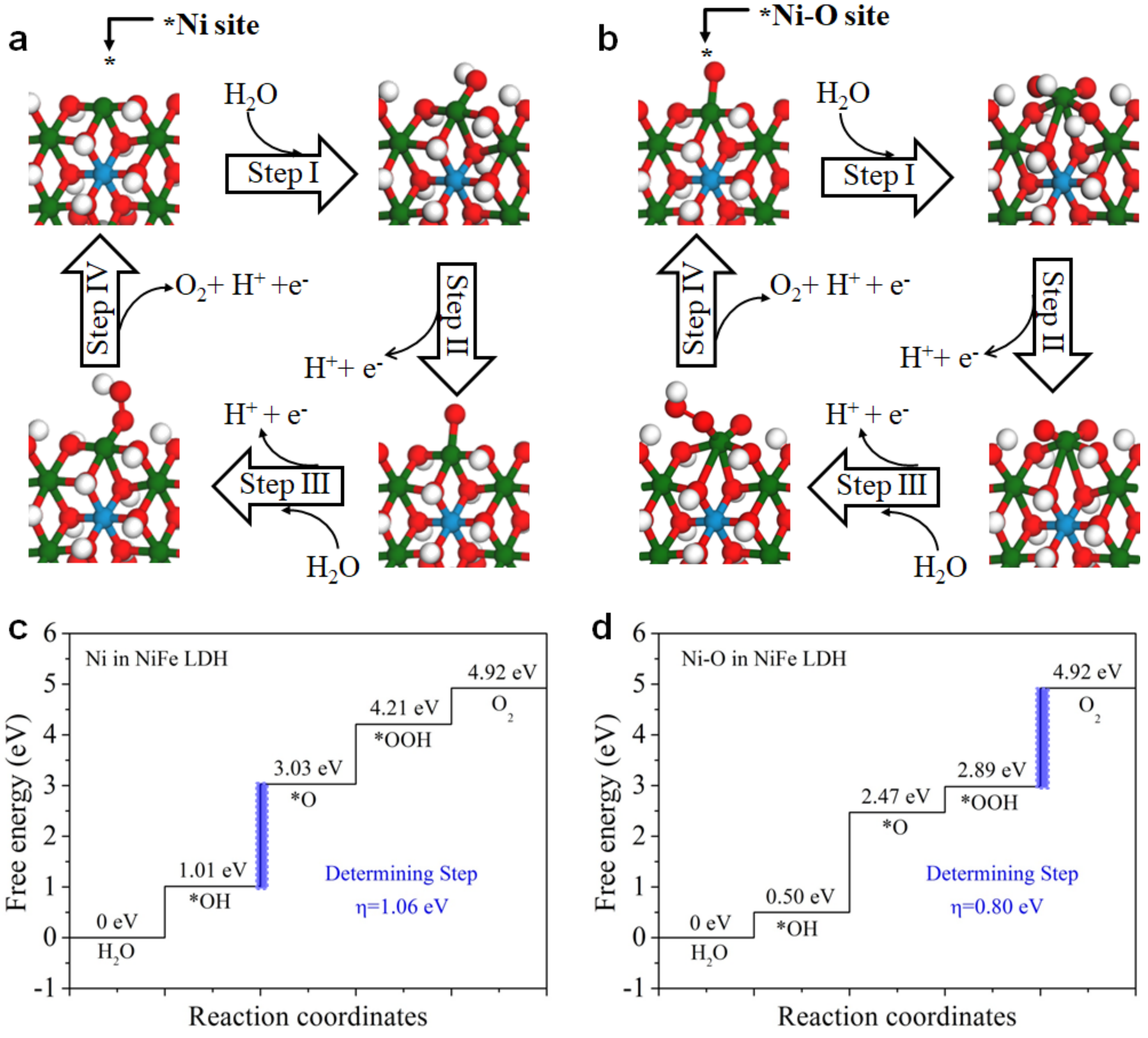

Supplementary Figure 35. Schematic illustration of the proposed OER mechanism for (a) Ni and (b) Ni–O sites in NiFe LDH. Gibbs free energy diagram for (c) Ni and (d) Ni–O sites in NiFe LDH. The lavender box step is the rate determining step.

Supplementary Table 1. EXAFS fitting parameters at the Fe, Ni and Ru K-edge various samples (S_0^2=0.74, 0.94, 0.83).

Catalysts	Path	C.N.	R (Å)	$\sigma^2 \times 10^3$ (Å^2)	ΔE (eV)	R factor
Fe foil	Fe-Fe	8	2.47	5.2	5.6	0.001
	Fe-Fe	6	2.84	8.4	3.9	
Fe_2O_3	Fe-O	5.9	1.99	12.9	-3.5	0.007
	Fe-Fe	5.5	2.99	6.4	3.3	
Ru/D-NiFe LDH	Fe-O	3.7	1.96	8.4	-5.6	0.014
	Fe-Ni	3.3	3.04	12.5	-1.8	
Ni foil	Ni-Ni	12	2.48	4.7	6.9	0.001
NiO	Ni-O	6.3	2.08	6.1	-1.9	0.004
	Ni-Ni	13.4	2.95	6.9	-3.7	
Ru/D-NiFe LDH	Ni-O	6.1	2.04	5.8	-6.8	0.014
	Ni-Fe	5.0	3.06	16.8	-5.3	
Ru foil	Ru-Ru	12	2.68	4.2	-5.2	0.003
RuO_2	Ru-O	5.9	1.98	2.2	-2.8	0.008
	Ru-Ru	6.2	3.12	8.0	-10.3	
	Ru-Ru	5.0	3.56	1.2	-3.2	
Ru_1/D-NiFe LDH	Ru-O	3.7	2.05	3.0	-3.0	0.002
	Ru-O-M	2.0	2.79	7.6	9.3	

[a]*N*: coordination numbers; [b]*R*: bond distance; [c]σ^2: Debye-Waller factors; [d]ΔE_0: the inner potential correction. *R* factor: goodness of fit.

Supplementary Table 2. Comparison of HER activity data for various catalysts.

Catalysts	Overpotential @10mA cm^{-2}	Tafel slope (mV/dec)	References
Co_1/PCN	89 mV	52	Nat. Catal. 2, 134 (2018)
C-$Ni_{1-x}O$	27 mV	36	Nat. Commun. 11, 590 (2020)
Ni-Fe NPs	46 mV	58	Nat. Commun. 10, 5599 (2019)
C-doped MoS_2	45 mV	46	Nat. Commun. 10, 1217 (2019)
$MoS_2/Co_9S_8/Ni_3S_2$/Ni	113 mV	85	J. Am. Chem. Soc. 141, 10417 (2019)
Co-NiS_2	80 mV	43	Angew. Chem. Int. Ed. 58, 18676 (2019)
V-Co_4N	37 mV	44	Angew. Chem. Int. Ed. 57, 5076 (2018)
Mo_3P/Mo	78 mV	43	Angew. Chem. Int. Ed. 57, 14139 (2018)
Cu/NiFe LDH	116mV	58.9	Energy Environ. Sci. 10, 1820 (2017)
Ni_5P_4-Ru	54 mV	52	Adv. Mater. 32, 1906972 (2020)
δ-FeOOH NSs/NF	108 mV	68	Adv. Mater. 30, 1803144 (2018)
ECM@Ru	63 mV	47	Adv. Energy Mater. 10, 2000882 (2020)
Ni-ZIF/Ni-B	67 mV	57	Adv. Energy Mater. 10, 1902714 (2019)
Mo-Co_9S_8@C	90 mV	34.6	Adv. Energy Mater. 10, 1903137 (2019)
NC/CuCo/$CuCoO_x$	112 mV	55	Adv. Funct. Mater. 28, 1704447 (2018)
$Co(S_xSe_{1-x})_2$	132mV	90	Adv. Funct. Mater. 27, 1701008 (2017)
Rh/NiFe LDH	81.3 mV	49	Nano Lett. 20, 136 (2020)
Ni_3N_{1-x}/NF	55 mV	54	Adv. Sci. 5, 1800406 (2018)
O-CoMoS	97 mV	70	ACS Catal. 8, 4612 (2018)
**Ru_1/D-NiFe LDH**	**18 mV**	**29**	**This work**

Supplementary Table 3. Comparison of TOF of HER catalysts in alkaline condition.

Catalysts	Overpotential (V)	TOF (H_2 S^{-1})	References
Co_1/PCN	0.05	0.22	Nat. Catal. 2, 134 (2018)
RuNi/CQDs	0.1	5.03	Angew. Chem. Int. Ed. 59, 1718 (2020)
Co-NiS_2	0.1	0.55	Angew. Chem. Int. Ed. 58, 18676 (2019)
$Mo_1N_1C_2$	0.1	0.465	Angew. Chem. Int. Ed. 56, 16086 (2017)
Ni_5P_4	0.1	0.79	Energy Environ. Sci. 8, 1027 (2015)
Ir/CON	0.025	0.2	Adv. Mater. 30, 1805606 (2018)
Ru@GnP	0.1	0.145	Adv. Mater. 30, 1803676 (2018)
$MoNi_4/MoO_{3-x}$	0.1	1.13	Adv. Mater. 29, 1703311 (2017)
Ir/NPs	0.04	0.07	Adv. Energy Mater. 8, 1801698 (2018)
Ru NPs	0.04	0.18	Adv. Energy Mater. 8, 1801698 (2018)
Rh NPs	0.04	0.39	Adv. Energy Mater. 8, 1801698 (2018)
NiMo alloy	0.1	0.05	ACS Catal. 3, 166-169 (2013)
**Ru_1/D-NiFe LDH**	**0.1**	**7.66**	**This work**

Supplementary Table 4. Comparison of OER activity data for various catalysts.

Catalysts	Overpotential @10mA cm^{-2}	Tafel slope (mV/dec)	References
Ni-NHGF	331 mV	63	Nat. Catal. 1, 63 (2018)
np-Ir/NiFeO	197 mV	29.6	Nat. Commun. 11, 2701 (2020)
Ru/CoFe-LDHs	198 mV	39	Nat. Commun. 10, 1711 (2019)
w-$Ni(OH)_2$	237 mV	33	Nat. Commun. 10, 2149 (2019)
e-ICLDH@GDY	216 mV	43.6	Nat. Commun. 9, 5309 (2018)
Ir/NiO	215 mV	38	J. Am. Chem. Soc. 142, 7425 (2020)
FeOOH(Se)/IF	287 mV	54	J. Am. Chem. Soc. 141, 7005 (2019)
CoO/Co_3O_4	260 mV	54	Angew. Chem. Int. Ed. 59, 6929 (2020)
CoFe LDH-Ar	266 mV	37.8	Angew. Chem. Int. Ed. 56, 5867 (2017)
$Ni/Ni(OH)_2$	270 mV	53	Adv. Mater. 32, 1906915 (2020)
W_2N/WC	320 mV	122.8	Adv. Mater. 32, 1905679 (2020)
HCM@Ni-N	304 mV	76	Adv. Mater. 31, 1904548 (2019)
δ-FeOOH NSs/NF	265 mV	69	Adv. Mater. 30, 1803144 (2018)
IrO_x/L-BN	285 mV	36.6	Adv. Energy Mater. 10, 1902521 (2020)
P-Co_3O_4	280 mV	51.6	Adv. Energy Mater. 10, 1902521 (2020)
Fe-CoOOH/G	330 mV	37	Adv. Energy Mater. 7, 1602148 (2017)
N-Fe_2PO_{5-x}-OT	235 mV	27.2	Adv. Functional Mater. 28, 1801397 (2018)
NiFe-OH-F	243 mV	42.9	Nano Lett. 19, 530 (2019)
**Ru_1/D-NiFe LDH**	**189 mV**	**31**	**This work**

Supplementary Table 5. Comparison of overall water splitting data for various catalysts.

Catalysts	Electrolyte	Cell Volatge (V)	References
$RuIrO_x$	1 M KOH	1.47 V	Nat. Commun. 10, 4875 (2019)
Ni-Fe NPs	1M KOH	1.47 V	Nat. Commun. 10, 5599 (2019)
CoMoNiS-NF	1M KOH	1.54 V	J. Am. Chem. Soc. 141, 10417 (2019)
R-$NiCo_2O_4$	1M KOH	1.61 V	J. Am. Chem. Soc. 140, 13644 (2018)
CoP/NCNHP	1M KOH	1.64 V	J. Am. Chem. Soc. 140, 2610 (2018)
CoMnO@CN	1M KOH	1.5 V	J. Am. Chem. Soc. 137, 14305 (2015)
RuCu NSs	1 M KOH	1.49 V	Angew. Chem. Int. Ed. 58, 13983 (2019)
Co/β-Mo_2C@N-CNTs	1 M KOH	1.64 V	Angew. Chem. Int. Ed. 58, 4923 (2019)
VOOH/NF	1 M KOH	1.62 V	Angew. Chem. Int. Ed. 56, 573 (2017)
Co_3O_4-MTA	1 M KOH	1.63 V	Angew. Chem. Int. Ed. 56, 1324 (2017)
Cu@NiFe LDH	1 M KOH	1.54 V	Energy Environ. Sci. 10, 1820 (2017)
EG/$Co_{0.85}$Se/NiFe LDH	1 M KOH	1.67 V	Energy Environ. Sci. 9, 478 (2016)
MoO_3/Ni-NiO	1 M KOH	1.55 V	Adv. Mater. 32, 2003414 (2020)
Ni/$Ni(OH)_2$	1 M KOH	1.59 V	Adv. Mater. 32, 1906915 (2020)
W_2N/WC	1 M KOH	1.58 V	Adv. Mater. 32, 1905679 (2020)
Cr-doped FeNi-P/NCN	1 M KOH	1.50 V	Adv. Mater. 31, 1900178 (2019)
δ-FeOOH NSs/NF	1 M KOH	1.62 V	Adv. Mater. 30, 1803144 (2018)
CoS_x@Cu_2MoS_4-MoS_2/NSG	1 M KOH	1.6 V	Adv. Energy Mater. 10, 1903289 (2020)
Ni-ZIF/Ni-B	1 M KOH	1.54 V	Adv. Energy Mater. 10, 1902714 (2019)
Mo-Co_9S_8@C	1 M KOH	1.56 V	Adv. Energy Mater. 10, 1903137 (2019)
Co@N-CS/N-HCP	1 M KOH	1.545 V	Adv. Energy Mater. 9, 1803918 (2019)

NiFe LDH@NiCoP	1 M KOH	1.57 V	Adv. Funct. Mater. 28, 1706847 (2018)
sNiCoP/NF	1 M KOH	1.58 V	Nano Lett. 16, 7718 (2016)
CoFe@NiFe LDH	1 M KOH	1.59 V	Appl. Catal. B 253, 131 (2019)
S-CoO_x	1 M KOH	1.63 V	Nano Energy 71, 104652 (2020)
$Zn_{1-x}Fe_x$-LDH	1 M KOH	1.62 V	Small 14, 1803638 (2018)
NiCoFeB	1 M KOH	1.81 V	Small 15, 1804212 (2019)
**Ru_1/D-NiFe LDH**	**1 M KOH**	**1.44**	**This work**

Supplementary Note 1. Calculation of the Ru mass activity.

In order to compare the Ru activities of Ru_1/D-NiFe LDH, their activity values have been normalized to Ru loading. The mass activities of HER and OER were evaluated at the overpotential of 100 mV and 240 mV, respectively. The details are as follows:

HER: at the overpotential of 100 mV

$$j_{mass}^{\mathrm{Ru}_1/\mathrm{D-NiFe\ LDH}} = \frac{j_{area}^{\mathrm{Ru}\,1/\mathrm{D-NiFe\ LDH}}\ (mA\ cm^{-2})}{mass_{\mathrm{Ru}}\ (mg\ cm^{-2})} = 14.65\ \mathrm{A\ mg^{-1}}$$

OER: at the overpotential of 240 mV

$$j_{mass}^{\mathrm{Ru}_1/\mathrm{D-NiFe\ LDH}} = \frac{j_{area}^{\mathrm{Ru}\,1/\mathrm{D-NiFe\ LDH}}\ (mA\ cm^{-2})}{mass_{\mathrm{Ru}}\ (mg\ cm^{-2})} = 11.98\ \mathrm{A\ mg^{-1}}$$

Supplementary Note 2. TOF measurement.

The TOF values can be obtained by utilizing the following equation.

$$TOF = \frac{\#\ Total\ Hydrogen\ Turn\ Overs\ per\ geometric\ area}{\#\ Active\ Sites\ per\ geometric\ area}$$

The number of total hydrogen turnovers was calculated from current density from polarization curve.

$$\#\ Total\ Hydrogen\ Turn\ Overs$$
$$= \left(j\frac{mA}{cm^2}\right)\left(\frac{1C\ s^{-1}}{1000\ mA}\right)\left(\frac{1\ mol\ e^-}{96485.3\ C}\right)\left(\frac{1\ mol\ H_2}{2\ mol\ e^-}\right)\left(\frac{6.022\ \times 10^{23}\ H_2\ molecules}{1\ mol\ H_2}\right)$$
$$= 3.12\ \times 10^{15}\ |\ j\ |\ \frac{H_2/s}{cm^2}\ per\ \frac{mA}{cm^2}$$

The number active sites in Ru_1/D-NiFe LDH were calculated from total mass of Ru on the electrode, assuming that all Ru atoms could contribute to the reaction process.

$$\#\ Active\ Sites$$
$$= \left(\frac{mass\ loading \times catalyst\ loading\ per\ geometric\ area\ (g/cm^2)}{Ru\ Mw\ (g/mol)}\right)\left(\frac{6.022\ \times 10^{23}\ Ru\ atoms}{1\ mol\ Ru}\right)$$
$$= \left(\frac{1.2wt\% \times 0.002\ (g/cm^2)}{101.1\ g/mol}\right)\left(\frac{6.022\ \times 10^{23}\ Ru\ atoms}{1\ mol\ Ru}\right)$$
$$=1.43\ \times 10^{17}\ Ru\ sites\ per\ cm^2$$

Finally, the current density from the polarization curves can be converted into TOF values according to the following equation.

$$TOF = \frac{3.12\ \times 10^{15}}{1.43\ \times 10^{17}} \times |\ j\ |\ = 0.0218|\ j\ |$$

Supplementary Note 3. XANES simulation.

The Ru K-edge theoretical XANES calculations were carried out with the FDMNES code in the framework of real-space full multiple-scattering (FMS) scheme using Muffin-tin approximation for the potential. The energy-dependent exchange-correlation potential was calculated in the real Hedin–Lundqvist scheme, and then the spectra convoluted using a Lorentzian function with an energy-dependent width to account for the broadening due both to the core–hole width and to the final state width. The Ru_1/D-NiFe LDH moieties were built based on the model of DFT calculation. To avoid artificial biases, all models were first optimized by DFT calculation. Satisfactory convergence for the cluster sizes had been achieved.
